# Epigenetic memories and the evolution of infectious diseases

**DOI:** 10.1038/s41467-021-24580-0

**Published:** 2021-07-13

**Authors:** David V. McLeod, Geoff Wild, Francisco Úbeda

**Affiliations:** 1 Centre D’Ecologie Fonctionnelle & Evolutive, CNRS, Montpellier, France; 2 Institute of Integrative Biology, ETH Zürich, Zürich, Switzerland; 3 Department of Applied Mathematics, The University of Western Ontario, London, ON Canada; 4 Department of Biology, Royal Holloway University of London, Egham, Surrey UK

**Keywords:** Evolutionary theory, Epigenetics, Infectious diseases

## Abstract

Genes with identical DNA sequence may show differential expression because of epigenetic marks. Where epigenetic marks respond to past conditions, they represent a form of “memory”. Despite their medical relevance, the impact of memories on the evolution of infectious diseases has rarely been considered. Here we explore the evolution of virulence in pathogens that carry memories of the sex of their previous host. We show that this form of memory provides information about the sex of present and future hosts when the sexes differ in their pathogen’s transmission pattern. Memories of past hosts enable the evolution of greater virulence in infections originating from one sex and infections transmitted across sexes. Thus, our results account for patterns of virulence that have, to date, defied medical explanation. In particular, it has been observed that girls infected by boys (or boys infected by girls) are more likely to die from measles, chickenpox and polio than girls infected by girls (or boys infected by boys). We also evaluate epigenetic therapies that tamper with the memories of infecting pathogens. More broadly, our findings imply that pathogens can be selected to carry memories of past environments other than sex. This identifies new directions in personalised medicine.

## Abstract (web summary)

The virulence of some infectious diseases seems to depend on the sex of the host the infection came from, as well as that of the current host. Here, McLeod et al. develop an epidemiological model to investigate the evolution of virulence when pathogens can retain epigenetic memories of their previous host.

## Introduction

**Fig. 1:**
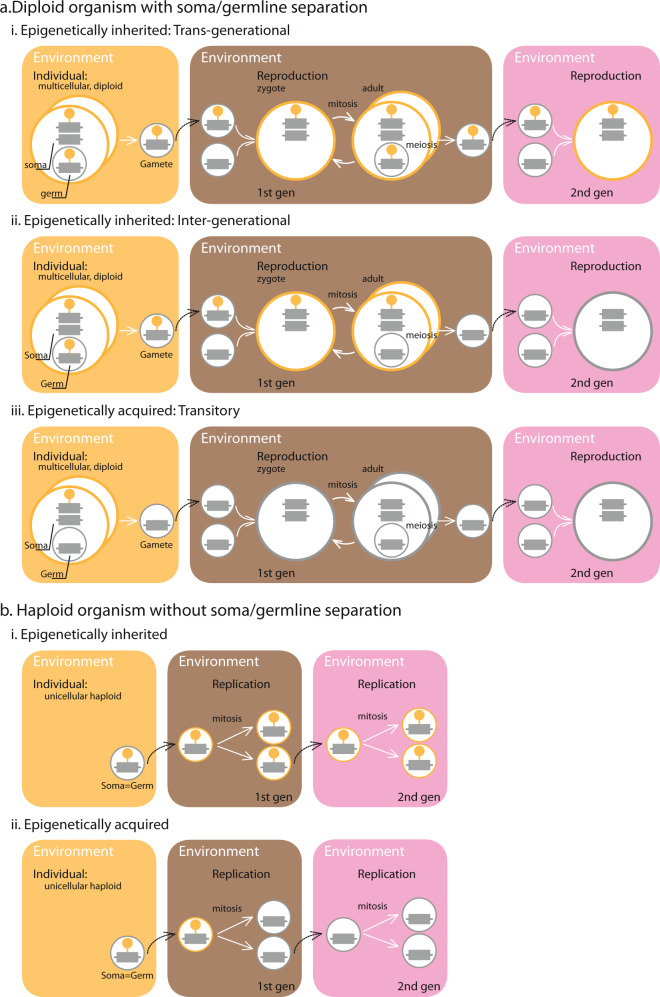
Schematic representation of the different types of epigenetic marks observed in different organisms. Panel **a** refer to complex organisms, that is diploid with germline-soma differentiation. Each sub-panel depicts a different type of epigenetic mark **a.i** trans-generational epigenetically inherited, **a.ii** inter-generational epigenetically inherited, and **a.iii** epigenetically acquired. Panel **b** refer to complex organisms that are haploid with no germline-soma differentiation. Sub-panel **b.i** depicts epigenetically inherited marks, while **b.ii** depicts epigenetically acquired marks.

In general, the term ‘epigenetics’ refers to the molecular mechanisms underlying the differential expression of genes with identical DNA sequences in response to environmental factors —for example, diet, stress, parental origin— thus resulting in different phenotypes (gene ‘plasticity’)^1,2^. Environmental factors leave marks that do not change the DNA sequence of the gene, for example, DNA methylation or histone modification^1–3^. Some of these ‘epigenetic marks’ are maintained during the lifetime of an individual (henceforth ‘epigenetically acquired’ marks) but they are not inherited from one generation to the next (Fig. 1a.iii). Other marks, however, are inherited across generations (henceforth ‘epigenetically inherited’ marks)^1,2^ (Fig. 1a.i-ii). There is abundant evidence of epigenetic inheritance in plants and mammals^2,4^. In plants, for example, the exposure of flax (*Linum usitatissimum*) to demethylating agents results in early flowering, a phenotype that continues to be observed in later generations when these agents are absent^5^. In mammals, the exposure of mice (*Mus musculus*) to early-life stress (maternal separation) results in depression, a phenotype that continues to be observed in descendants that were not separated from their mothers^6,7^. Epigenetic inheritance thus opens up the fascinating possibility that genes have ‘memories’ of past environmental conditions that, in turn, affect their expression even after conditions have changed.

Due to its medical and developmental implications, epigenetic inheritance has received a lot of attention that has advanced our knowledge of phenomena like cancer and foetal growth^8,9^. Despite the abundant attention that epigenetic inheritance has received, to our knowledge memories have not been considered when studying the epidemiology and evolution of infectious diseases. This oversight is not justified from a scientific-interest perspective: epigenetic inheritance has the potential to affect a wide array of pathogen phenotypes. The oversight is also not justified from a medical perspective: if we better understand the forces that act to shape pathogens, then we can possibly devise better strategies to treat the infections they create. Finally, the oversight is not justified from an empirical perspective: there is abundant evidence of epigenetic marks established through exposure to environmental conditions that regulate the expression of genes underpinning virulence or transmission^1,3,10–18^. Furthermore, some of these epigenetic marks are inherited across multiple generations of the pathogen, e.g., marks carried by the EBV virus, *Salmonella enterica*, *Candida albicans* and *Plasmodium falciparum*^1,3,11,18^. In the case of *P. falciparum*, exposure to the antibiotic blasticidin, leads this pathogen to epigenetically adjust the expression profile of its *var*-family genes to avoid the action of the antibiotic itself; these epigenetic marks are transmitted to multiple generations of pathogens after the antibiotic has disappeared^10,19^. Here we work towards filling this gap by considering epigenetic memories in the evolution of infectious pathogens.

**Fig. 2:**
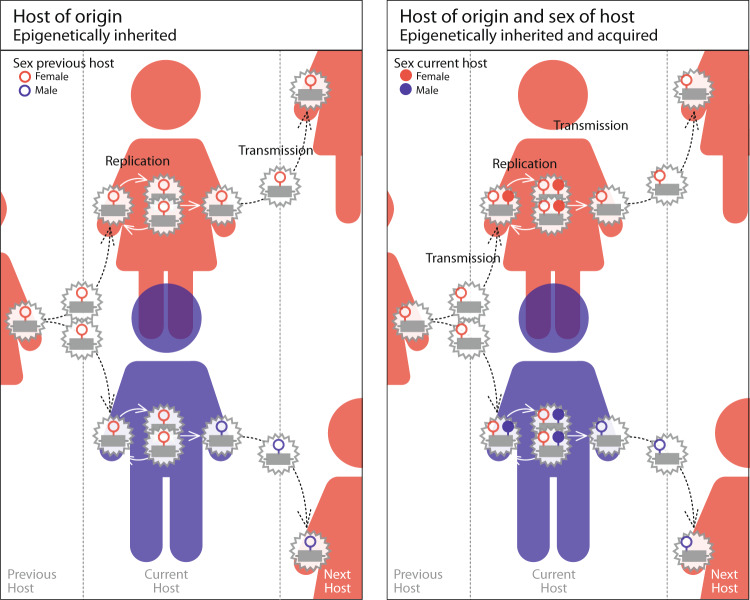
Cycle of epigenetic marks on pathogens. The first panel represents the cycle of epigenetically inherited marks on pathogens, that is marks underpining origin-specific virulence. The second panel represents the cycle of epigenetically inherited and acquired marks on pathogens, that is marks underpining origin-&-sex-specific virulence.

We advance theory by studying the evolution of virulence in pathogens that can remember the sex of the host from which they came (henceforth, ‘origin-specific virulence’) (Fig. 2a). We outline conditions under which natural selection favours pathogens that retain the memory of the sex of the host they originated from over those that do not. Furthermore, we make testable predictions about the greater virulence of pathogens inherited from one sex as opposed to the other. In addition, we investigate when pathogens that can condition their virulence on the sex of their previous host will be selected to add information regarding the sex of their current host (henceforth, ‘origin-&-sex-specific virulence’) (Fig. 2b). We make explicit predictions about the greater virulence of pathogens originating from and infecting the same sex, as opposed to originating from and infecting the opposite sex. By providing testable predictions we aim to motivate experimental work that test epigenetic memories in pathogens.

While there is abundant evidence that pathogens can retain memories of past environments^1,3,11,18^, we are not aware of experiments testing whether pathogens retain the sex of their previous host. However, information on the sex of the previous carrier is widespread in genes of plants and mammals that are imprinted^20–22^. Parallels on information acquisition can be drawn between gametic cells proliferating in female and male somatic environments and pathogens multiplying in females and male hosts. If genes in gametic cells are able to acquire information on the sex of their current carrier and pass it on to genes in the next carrier (imprinted genes) it does not seem too far fetched to consider genes in pathogens that are able to acquire information on the sex of their current host and pass it on to pathogenic genes in the next host (in particular when it has been shown that pathogens can acquire information on their current environment and maintain it through division^1,3,11,18^).

Our results can explain the puzzling observations of virulence patterns in measles, chickenpox and polio wherein those with infections contracted from same-sex individuals develop a less virulent infection compared to those infected by the opposite sex^23–26^. That, in developing countries, girls infected with measles by boys are more likely to die from the infection than girls infected by girls, is a well-established result that remains poorly understood^23,24,27^. What is interesting about this pattern is that it cannot be explained by differences in virulence between girls and boys (i.e., due to differences between the sexes in their immune system) as differences between the sexes will affect equally infections received from girls and boys. Here we argue that pathogens with epigenetic memories of the sex of the host from which they came, can explain the complex patterns of virulence found in measles, chickenpox and polio. Finally, we explore the implications of our findings for the treatment of infectious diseases. In particular, we predict when the use of drugs that erase some epigenetic memories in pathogens will reduce their virulence.

## Results

### Infectious-disease dynamics

**Fig. 3:**
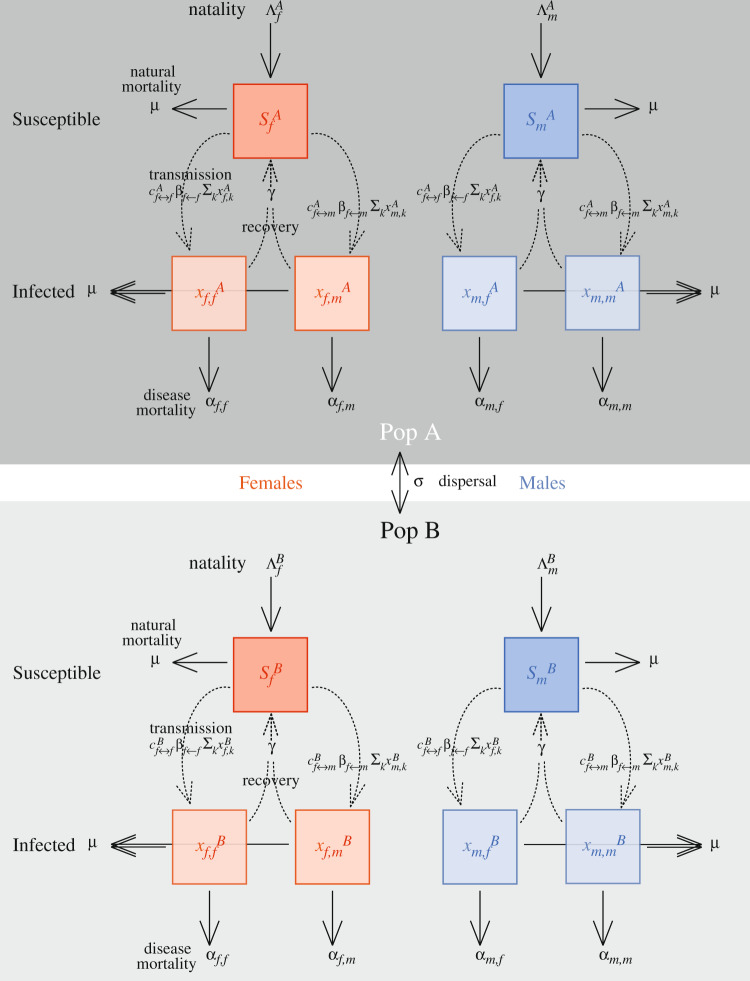
Flow diagram of two subpopulation epidemiological model. More details can be found in the main text and Note 1 of the Supplementary Information.

We consider a structured host population consisting of two possible host types, female (type *j* = *f*) and male (type *j* = *m*). A host of a given sex, *j*, infected with a pathogen that most recently originated from a sex-*k* host, recovers from its infection at rate *γ*, dies from causes unrelated to the infection at rate *μ* (natural mortality) and dies from causes related to the infection at a rate *α*_*j*,*k*_ (virulence) (see Fig. 3).

We model transmission using a law of mass-action with rate constant *β*_*i*←*j*_, where *i* in this context refers to the sex of the susceptible host and *j* refers to the sex of the infective host. We make the standard assumption that there is a trade-off between virulence and transmission^28–31^. This trade-off could be mediated by the rate of pathogen replication within an infected host^28–31^. For example, a greater replication rate could increase disease transmission but would also increase host mortality. For clarity, we will use ${\beta }_{i\leftarrow j}({\alpha }_{j,k})=\frac{{\alpha }_{j,k}}{{\alpha }_{j,k}+{\theta }_{i\leftarrow j}}$, where *θ*_*i*←*j*_ is a positive parameter that controls how quickly the transmission benefits of higher virulence saturate.

Hosts also belong to one of two subpopulations, *ℓ* = *A* and *ℓ* = *B*, and individual hosts move between these at a per-capita rate *σ*. Subpopulations differ with respect to the pattern of contact between hosts and/or the influx susceptible hosts. We use ${c}_{i\leftrightarrow j}^{\ell }={c}_{j\leftrightarrow i}^{\ell }$ to denote the probability with which an interaction between a sex-*i* host and sex-*j* host occurs in subpopulation *ℓ*. In addition, we use ${\Lambda }_{j}^{\ell }$ to denote the total rate at which sex-*j* susceptible individuals are recruited to subpopulation *ℓ* (e.g., through birth or immigration) (see Fig. 3). If ${c}_{i\leftrightarrow j}^{A}={c}_{i\leftrightarrow j}^{B}$ and ${\Lambda }_{j}^{A}={\Lambda }_{j}^{B}$ for all *i*, *j*, then the two-subpopulation model collapses to a one-population model. We will consider the one- and two-subpopulation models separately.

Let ${S}_{j}^{\ell }$ and ${x}_{j,k}^{\ell }$ denote the density of susceptible sex-*j* hosts, and infected sex-*j* hosts who contracted the infection from a sex-*k* host, respectively, in subpopulation *ℓ* at time *t*. Then the description above gives rise to the following system of twelve differential equations:

1 $${\dot{S}}_{i}^{\ell } 	={\Lambda }_{i}^{\ell }-\mu {S}_{i}^{\ell }-{S}_{i}^{\ell }\ {\sum }_{j}{c}_{i\leftrightarrow j}^{\ell }{\sum }_{k}{\beta }_{i\leftarrow j}({\alpha }_{j,k})\ {x}_{j,k}^{\ell }+\gamma \ {\sum }_{j}{x}_{i,j}^{\ell }+\sigma ({S}_{i}^{\neg \ell }-{S}_{i}^{\ell }),\\ {\dot{x}}_{j,k}^{\ell } 	={S}_{j}^{\ell }{c}_{j\leftrightarrow k}^{\ell }{\sum }_{i}{\beta }_{j\leftarrow k}({\alpha }_{j,k})\ {x}_{k,i}^{\ell }-({\alpha }_{j,k}+\mu +\gamma ){x}_{j,k}^{\ell }+\sigma ({x}_{j,k}^{\neg \ell }-{x}_{j,k}^{\ell }),$$

where dots indicate differentiation with respect to time, where *ℓ*, ¬*ℓ* ∈ {*A*, *B*} with ¬*ℓ* ≠ *ℓ*, and where *i*, *j*, *k* ∈ {*f*, *m*}.

### Evolutionary dynamics of pathogen virulence

We study the evolutionary dynamics of pathogen virulence using an invasion analysis^32,33^. This analysis considers a rare mutant pathogen that expresses a novel virulence phenotype as it competes against an established resident strain. It relies on the assumption that the dynamics described in (1) have brought the global population very close to an equilibrium at which the resident strain is maintained in an endemic state.

Typically an invasion analysis yields a measure of the invasion fitness of a rare mutant. In this case, the measure would reflect the expected number of secondary infections created by a mutant expressing virulence phenotype ${\tilde{\alpha }}_{j,k}\ne {\alpha }_{j,k}$. Rather than working directly with this measure, we opt to investigate its first derivative, i.e. the selection gradient:

2 $${\lambda }_{j,k}^{\prime}({\alpha }_{j,k})=\mathop{\sum}\limits_{\ell =A,B}{\bar{x}}_{j,k}^{\ell }{ \Big[ \mathop{\sum}\limits_{i = f,m}{v}_{i,j}^{\ell }\underbrace{{c}_{i\leftrightarrow j}^{\ell }{\bar{S}_{i}^{\ell }\frac{d{\beta }_{i\leftarrow j}({\tilde{\alpha }}_{j,k})}{d{\tilde{\alpha }}_{j,k}}}}_{({\rm{a}})}-{v}_{j,k}^{\ell }\underbrace{{\frac{d{\tilde{\alpha }}_{j,k}}{d{\tilde{\alpha }}_{j,k}}}}_{({\rm{b}})}}\Big]_{{\tilde{\alpha }}_{j,k} = {\tilde{\alpha }}_{j,k}}$$

where ${v}_{i,j}^{\ell }$ is the reproductive value of a *i* ← *j* infection currently found in subpopulation *ℓ* and overbars indicate variables at equilibrium (e.g., ${\bar{x}}_{j,k}^{\ell }$ is ${x}_{j,k}^{\ell }$ at equilibrium). Term (a) in Eq. (2) expresses the rate at which secondary *i* ← *j* infections in subpopulation *ℓ* rise with increasing mutant virulence. That is the upside of increased virulence. Term (b), on the other hand, represents the rate at which host mortality goes up as mutant virulence increases and captures the downside. By weighting (a) and (b) by reproductive value, Eq. (2) captures the long-term evolutionary significance of small changes in mutant virulence. Overall, the selection gradient, ${\lambda }_{j,k}^{\prime}({\alpha }_{j,k})$, gives the direction of travel through trait space that produces the greatest instantaneous increase in mutant fitness. When this quantity is positive (resp. negative), selection favours an increase (resp. decrease) *α*_*j*,*k*_.

The selection gradient revealed by our analysis, along with any constraints owing to a pathogen’s inability to adjust based on the sex of its host or its host of origin, establishes the evolutionary trajectory for each resident virulence trait *α*_*j*,*k*_. We expect to find an ‘evolutionarily stable’ (ES) virulence trait, denoted ${\alpha }_{j,k}^{* }$, at the end of a given trajectory. We identify these ES values computationally using an iterative numerical procedure (see “Methods” and Note 1 of the Supplementary Information).

It is not difficult to intuit that the evolutionary dynamics associated with (2) could give rise to ES levels of virulence that depend on the sex of a pathogen’s current host (current sex *j*). Given that transmissibility *β*_*i*←*j*_ explicitly depends on *j*, we expect the nature of the virulence-transmission trade-off to change depending on the sex of the host in which a strain is currently found; thus, the optimal balance struck between transmissibility and virulence follows suit in a sex-specific manner. This basic point can be derived from models with different types of hosts when assuming that the types considered are female and male hosts^34^. Interestingly, when transmissibility does not explicitly depend on the sex of the current host (infectivity), but does explicitly depend on the sex of the future host (future sex *i*) (susceptibility), natural selection does not favour the evolution of sex-specific virulence. When transmissibility does not explicitly depend on either the sex of the current or future host, natural selection favours the evolution of sex-specific virulence when vertical transmission is considered^35^.

It may be more difficult to intuit that evolutionary processes like the one suggested by (2) would predict ES virulence traits that depend on the sex of the host from which a given infection was acquired (sex of origin). This is especially true because, in the model we present here, sex of origin does not explicitly affect the transmission-virulence trade-off. We relax this assumption in the Supplementary Information (Note 1) for completeness. Nevertheless, adjusting virulence in the sex of origin can be advantageous when (i) the trade-off is affected by the sex of the susceptible host (susceptibility), and (ii) a sufficiently strong correlation exists between the sex of the host of origin and the sex of the host in which the next infection is likely to become established. As we detail below, the former requirement can be established by assuming one sex is more resistant to infection than the other, and the latter requirement can be established in the two-subpopulation setting we have here.

Despite the possibly complicated relationship between transmissibility and virulence, when describing our results we will compare transmissibilities (and only transmissiblities) that arise using a benchmark level of virulence. This benchmark, denoted *α*_bm_, reflects the predictions made by our model under the assumption of no pathogen plasticity. By evaluating *β*_*i*←*j*_ at the benchmarked *α* value, we can highlight the transmission differences that precede the origin of pathogen plasticity. To be clear, *β*_*i*←*j*_ should be henceforth understood as *β*_*i*←*j*_(*α*_bm_).

In the Supplementary Information (Note 2), we provide more details on our evolutionary analysis, including explicit calculations using the selection gradient; here we focus upon the key results. In particular, we address two questions: i. When does origin-specific or origin-&-sex-specific virulence evolve? ii. When do we expect to observe greater virulence of pathogens with a particular origin or a particular origin in a particular sex? We pay attention to contrasting patterns of virulence exhibited by infections transmitted between same-sex individuals against patterns exhibited by infections transmitted between opposite-sex individuals. This contrast is deserving of special attention as it is related to complex patterns of virulence in measles, chickenpox and polio—patterns that cannot be explained by recourse to sex-specific virulence.

### Origin-specific virulence: no population structure

In this section and the next, we explore the evolution of virulence when pathogens have epigenetically inherited information about the sex of the host from which they came, denoted *α*_•,*k*_. Henceforth, bullets represent a lack of dependency of the variable on the term they replace.

**Fig. 4:**
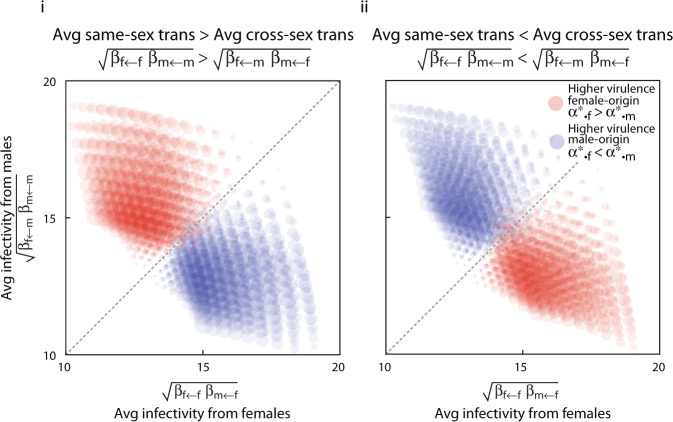
Evolution of origin-specific virulence with different infectivity and susceptibility between the sexes. On each sub-panel, the difference between evolutionarily stable virulence of female- and male-acquired infections are plotted as circles whose area scales with the extent of the difference. Circles are centred according to the average infectivity of females, defined as $\sqrt{{\beta }_{m\leftarrow f}{\beta }_{f\leftarrow f}}$, and average infectivity of males, defined as $\sqrt{{\beta }_{f\leftarrow m}{\beta }_{m\leftarrow m}}$. In subpanel **i**, average transmissibility between individuals of the same sex, defined as $\sqrt{{\beta }_{f\leftarrow f}{\beta }_{m\leftarrow m}}$, is higher than the average transmissibility between individuals of the opposite sex, defined as $\sqrt{{\beta }_{f\leftarrow m}{\beta }_{m\leftarrow f}}$. In sub-panel **ii**, the average transmissibility between individuals of the same sex is lower than the average transmissibility between individuals of the opposite sex. In both sub-panels, the results are based on a one-population model in which there are no sex-specific differences in background mortality, no possibility of recovery, and no difference in the influx of sexes to the population. Parameters (see Supplementary Information Note 1 and Available Code): *c-values all 1, m* = 35, *ρ* = 0.5, *μ* = 0.5, *γ* = 0, *θ*_*i*←*j*_ ranged from 0.5 to 1.5 and all combinations were considered (9^4^different combinations).

Origin-specific virulence can evolve when transmissibility depends on both the sex in which the pathogen currently resides and the sex that follows (see Note 5 of the Supplementary Information and Fig. 4). In this circumstance, two patterns emerge. If the average same-sex transmissibility exceeds the average cross-sex transmissibility, then greater virulence occurs in infections originating from the sex with greater average infectivity (if $\sqrt{{\beta }_{f\leftarrow f}{\beta }_{m\leftarrow m}}\;> \; \sqrt{{\beta }_{f\leftarrow m}{\beta }_{m\leftarrow f}}$, then ${\alpha }_{\bullet ,k}^{* }\; > \; {\alpha }_{\bullet ,\neg k}^{* }$ when $\sqrt{{\beta }_{f\leftarrow k}{\beta }_{m\leftarrow k}}\; > \; \sqrt{{\beta }_{f\leftarrow \neg k}{\beta }_{m\leftarrow \neg k}}$; Fig. 4i). By contrast, if average cross-sex transmissibility exceeds same-sex transmissibility, then the pattern is reversed: pathogen originating from the sex with the lower infectivity are more virulent (Fig. 4ii).

The intuition behind this result is that a pathogen’s origin provides information about the sex of the current host. Virulence then evolves to be lower in the sex that can infect more readily. For example, if the average cross-sex transmissibility is higher than the average same-sex transmissibility, then a pathogen with female origin is more likely to be infecting a male. In this case, when the average transmissibility from a sex (i.e. the infectivity of that sex) is higher in males, pathogens are selected to evolve lower virulence in males which corresponds to lower virulence when of female origin. In this scenario, origin-specific marks provide information about the sex of the current host. If reliable information about the sex of the current host were available, selection would favour the evolution of sex-specific virulence over origin-specific virulence. If a pathogen can gain information on the sex of its current host immediately after infection (or shortly after), it would not be selected to keep memories. However, if it takes the pathogen some time to gain this information, it will be selected to keep memories.

### Origin-specific virulence: population structure

**Fig. 5:**
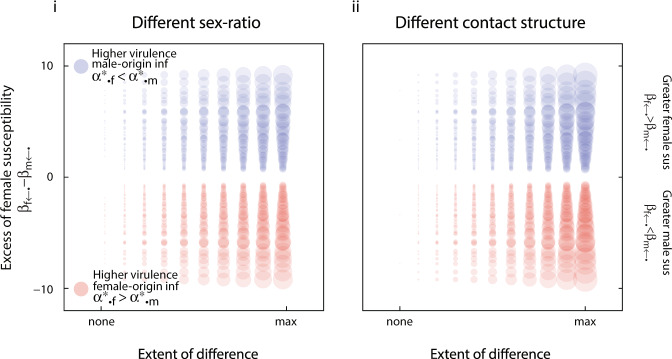
Evolution of origin-specific virulence with different subpopulations and different susceptibility between the sexes. On each sub-panel the difference between evolutionarily stable virulence associated with female- and male-acquired infections are plotted as circles whose area scales with the extent of the difference. Circles are centred according to the extent of the difference between subpopulations and the excess female susceptibility, defined as *β*_*f*←•_ − *β*_*m*←•_. There are no sex-specific differences in background mortality, there was no possibility of recovery, and there was only weak mixing of populations (*σ* = 0.25). In sub-panel **i**, the subpopulations differ only in their sex-ratio (controlled through the influx of susceptible hosts), from a scenario with no difference (indicated as `none') to one in which one subpopulation is strongly female biased while the other is strongly male biased (indicated as **‘**max**’**). In sub-panel **ii**, the subpopulations differ in their contract structure, from a scenario with no difference (**‘**none**’**) to one in which in subpopulation *A*, contacts are exclusively same-sex (${c}_{i\leftrightarrow j}^{A}={c}_{j\leftrightarrow j}^{A}$), while in subpopulation *B*, contacts are exclusively cross-sex (${c}_{i\leftrightarrow j}^{B}={c}_{\neg j\leftrightarrow j}^{B}$).

We now assume transmission depends only on the sex to which the pathogen is transmitted (susceptibility of a sex), *β*_*i*←*j*_ = *β*_*i*←•_. Here, origin-specific virulence evolves as long as subpopulations differ with respect to the sex-specific influx of new susceptible individuals (${\Lambda }_{j}^{A}\;\ne\; {\Lambda }_{j}^{B}$), or with respect to contact structure (${c}_{i\leftrightarrow j}^{A}\; \ne\; {c}_{i\leftrightarrow j}^{B}$) (Fig. 5 and Supplementary Information Note 4). Most notably, when sex-*k* is less susceptible to infection, the virulence of pathogens with sex-*k* origin evolves to be greater (if *β*_*k*←•_ < *β*_¬*k*←•_, then ${\alpha }_{\bullet ,k}^{* }\;> \; {\alpha }_{\bullet ,\neg \ k}^{* }$; Fig. 5).

The intuition behind this result is that the differences between subpopulations establishes a positive correlation between the sex of the host the pathogen will infect, and the sex of the host from which the pathogen originated. Virulence evolves to be lower when an infection originates from the sex that can be infected more readily. For example, if subpopulation A shows a higher rate of same-sex contacts and subpopulation B a higher rate of cross-sex contacts, a female-origin pathogen in either of these populations is likely to end up infecting a female next. When the susceptibility is higher in males, pathogens are selected to evolve higher virulence when of female origin. In this scenario origin-specific marks provide information about the sex of the next host. In this case, even if information about the sex of the current host were available, selection favours the evolution origin-specific virulence over sex-specific virulence.

### Origin-&-sex-specific virulence

So far we have explored the evolution of virulence when pathogens can only make use of inherited information. Here we extend our previous analysis to include pathogens that can adjust their virulence in response to inherited information about the sex of the host they came from, and acquired information about the sex of the host in which they currently reside, denoted *α*_*j*,*k*_. This extension is motivated by the complex virulence patterns observed in measles, chickenpox, and polio^25,36^. These infections result in greater mortality in girls infected by boys and in boys infected by girls^25,36^, a pattern that cannot be explained by invoking origin-specific or sex-specific virulence alone. In particular, sex-specific virulence could explain greater mortality arising from infections occurring in a given sex, and origin-specific virulence could explain greater mortality in infections originating from a given sex, but neither alone can yield the observed virulence patterns. Thus, it takes origin-&-sex-specific virulence to explain the reported interaction effects. What’s more, shifting the focus from pathogens to hosts, these patterns cannot be readily explained by sex-specific differences in the immune response or gene expression profiles alone. These differences would explain sex-specific virulence but not those differences seen in measles, chickenpox and polio.

In the absence of population structure, origin-&-sex-specific virulence will not evolve as information about the sex of the host of origin provides no adaptive value beyond information about the sex of the current host (i.e., sex-specific virulence).

The two-subpopulations model expands the scope for the evolution of origin-&-sex-specific virulence. In this section, we explore two ways in which this expansion can occur.

Firstly, in the presence of population structure, origin-&-sex-specific virulence can evolve even when transmissibility is the same for all host types (that is, *β*_*i*←*j*_ = *β*_•←•_). In this case, however, subpopulations must differ with respect to the sex-specific influx of susceptible hosts (${\Lambda }_{j}^{A}\; \ne\; {\Lambda }_{j}^{B}$), and the pattern of contact between host types (${c}_{i\leftrightarrow j}^{A}\; \ne\; {c}_{i\leftrightarrow j}^{B}$). Origin-&-sex-specific virulence is favoured, then, because it is an indirect response to the conditions the pathogen is likely to encounter in the future.

**Fig. 6:**
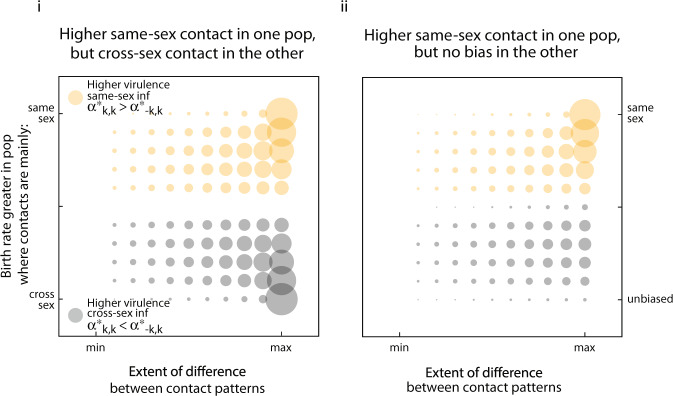
Evolution of origin-&-sex-specific virulence when subpopulations differ in contact structure and population size. The difference between average virulence associated with infections acquired from same-sex transmission, defined as ${\bar{\alpha }}_{kk}^{* }=({\alpha }_{11}+{\alpha }_{22})/2$, and those acquired from cross-sex transmission, defined as ${\bar{\alpha }}_{\neg kk}=({\alpha }_{12}+{\alpha }_{21})/2$, is plotted here as circles whose area scales with the extent of the difference. Circles are centred according to the relationship between the contact patterns that exist in the respective subpopulations, and the extent of the bias in birth rate between subpopulations. These numerical results assume no sex-specific differences in transmissibility or susceptibility (*β*_•←•_), no sex-specific background mortality, no possibility of recovery, and weak mixing of populations (*σ* = 0.25). In sub-panel **i**, there is higher same-sex contact in one subpopulation but lower in the other. As the difference with respect to contact patterns between the populations increases, contacts in one subpopulation become more strongly biased toward same-sex interactions while those in the other subpopulation become equally biased but toward cross-sex interactions. Opportunities for the creation of new infections can be strongly associated with same-sex interactions when the influx of new susceptible hosts through birth strongly favours the population with that contact pattern. Birth rates may also be greater in the subpopulation associated with cross-sex interactions, in which case the negative consequences of pathogen virulence are lower there and greater cross-sex virulence evolves. In sub-panel **ii**, there is higher same-sex contact in one population but no bias in the other. This assumes no bias in the same-sex vs cross-sex pattern of contact in one of the two subpopulations. In this case the opportunity to create new infections ranges between same-sex bias and no bias as the influx of new susceptible hosts shifts through births from favouring one subpopulation to favouring the other.

As an example, consider a case in which contacts in one subpopulation tend to occur more frequently between same-sex individuals (${c}_{j\leftrightarrow j}^{A}\; > \; {c}_{j\leftrightarrow \neg j}^{A}$), whereas contacts in the other subpopulation tend to be between individuals of the opposite sex (${c}_{j\leftrightarrow j}^{A}\; <\; {c}_{j\leftrightarrow \neg j}^{A}$). Suppose further that opportunities to create new infections are more abundant in the former subpopulation because birth rates there are greater (${\Lambda }_{j}^{A}\; > \; {\Lambda }_{j}^{B}$). Under these conditions, we find higher virulence from infections acquired from same-sex individuals on average, meaning $({\alpha }_{f,f}^{* }+{\alpha }_{m,m}^{* })\ > \ \frac{1}{2}({\alpha }_{f,m}^{* }+{\alpha }_{m,f}^{* })$ (Fig. 6).

The intuition behind this result rests on a two-part rationale. First, when births balance deaths on a global scale, as they do in our model at equilibrium, a higher birth rate in one subpopulation disproportionately buffers the negative consequences of virulence there. As a result, there is less disincentive to be virulent in the population where same-sex contacts dominate. Second, knowing that the sex of the host of origin matched (resp. did not match) the sex of the current host would suggest to a pathogen that the consequences of virulence are less (resp. more) dire than they might be otherwise.

Second, the evolution of origin-&-sex-specific virulence can be supported in the presence of sex-specific transmissibilities as well. In order for this virulence pattern to evolve, transmissibilities must show both sex-specific susceptibility and sex-specific infectivity. If transmissibilities are sex-specific, though, we can relax conditions on subpopulations, insisting that they only differ with respect either the sex-specific in susceptible hosts, or the pattern of contacts between host types. As before, origin-&-sex-specific virulence is favoured because it is an indirect response to the conditions the pathogen is likely to encounter in the future.

**Fig. 7:**
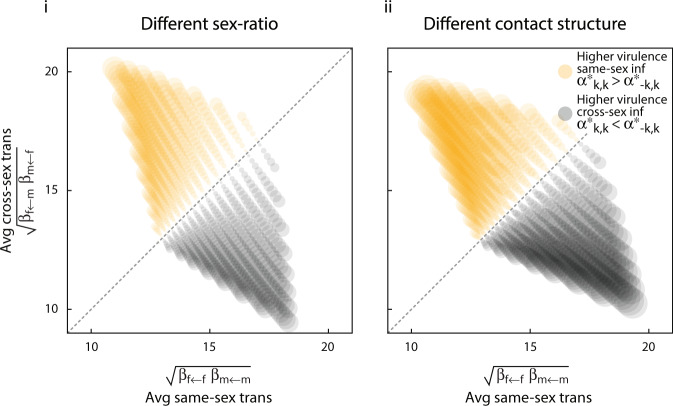
Evolution of origin-&-sex-specific virulence when subpopulations differ in same-sex and cross-sex transmission. The difference between average virulence associated with infections acquired from same-sex transmission and those acquired from cross-sex transmission are plotted as circles whose area scales with the extent of the difference itself. Circles are centred according to average same-sex transmissibility and average cross-sex transmissibility. We assume no sex-specific differences in background mortality, no possibility of recovery, and weak mixing of populations (*σ* = 0.1). In sub-panel **i**, the subpopulations differ in their sex-ratio such that the influx of sexes to each subpopulation is biased in exactly opposite directions, but contact structure does not differ between populations (${c}_{i\leftrightarrow j}^{A}={c}_{i\leftrightarrow j}^{B}=1$). In sub-panel **ii**, the subpopulations differ in their contact structure. Influx of sexes to each population is unbiased, but same-sex contact is more common in subpopulation *A* (${c}_{j\leftrightarrow j}^{A}\; > \; {c}_{\neg j\leftrightarrow j}^{A}$), while cross-sex contact is more common in subpopulation *B* (${c}_{j\leftrightarrow j}^{B}\; <\; {c}_{\neg j\leftrightarrow j}^{B}$).

In general, when average same-sex transmissibility is higher, then infections currently found in one sex but originating from the opposite sex show greater average virulence (if $\sqrt{{\beta }_{f\leftarrow f}{\beta }_{m\leftarrow m}}\; > \; \sqrt{{\beta }_{f\leftarrow m}{\beta }_{m\leftarrow f}}$, then $\frac{1}{2}({\alpha }_{f,f}^{* }+{\alpha }_{m,m}^{* })\; <\; \frac{1}{2}({\alpha }_{f,m}^{* }+{\alpha }_{m,f}^{* })$; Fig. 7).

## Discussion

**Fig. 8:**
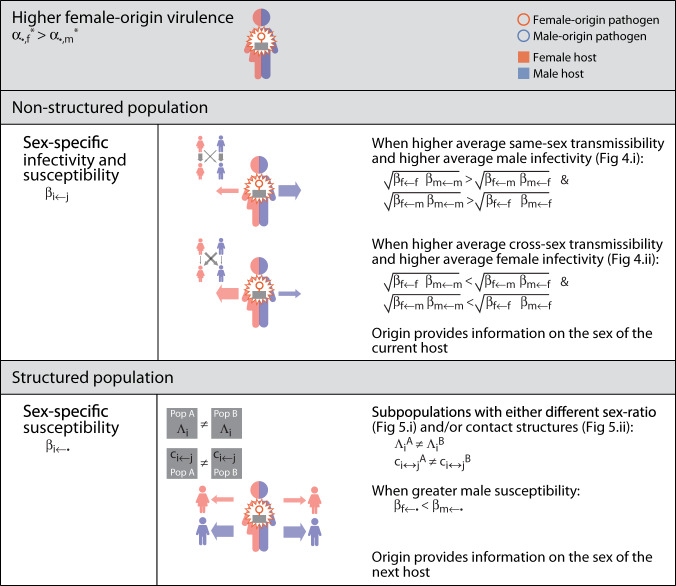
Summary of results regarding the evolution of origin-specific virulence. We focus on the case when female-origin virulence will be greater than male-origin one. Each row indicates a set of different assumptions regarding the population structure and pathogen’s transmissibility.

**Fig. 9:**
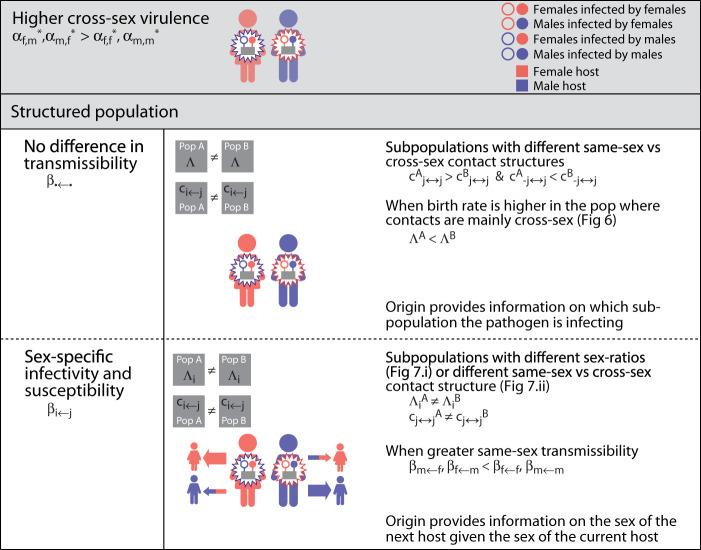
Summary of results regarding the evolution of origin-&-sex-specific virulence. We focus on the case when cross-sex virulence will be greater than same-sex one. Each row indicates a set of different assumptions regarding the population structure and pathogen’s transmissibility.

Our research shows that genes in pathogens can be selected to retain epigenetic memories of the sex of the host from which the infection originated. Our research also shows that genes in pathogens can be selected to combine epigenetic memories of the sex of the host from which the infection originated with information on the sex of the current host. Pathogens are selected to retain epigenetic memories when they convey reliable information about: the past, present, and future sex of the host they infect. Epigenetic memories about a past host can provide the pathogen: (i) information about opportunities for transmission that depend on the host they originated from; (ii) information about the sex of the current host (Fig. 8); (iii) information about the sex of future hosts (Fig. 9). Epigenetic memories about a past host that evolve to provide indirect information about the sex of the current host will be out-competed by direct information on the sex of the current host. On the contrary, epigenetic memories about a past host that evolve to provide information about the sex of future hosts (e.g., due to asymmetries in contact networks, or difference density of susceptibles) will out-compete information on the sex of the current host.

From an abstract perspective, our findings relate to previous work on the evolution of epigenetic cues in heterogeneous environments^37–41^. When the environment experienced by the individual is heterogeneous, epigenetic memories can evolve when they contain information about the current environment (e.g., strong environmental autocorrelation or limited migration) that could not be obtained otherwise (e.g., due to time lags, environmental noise/variability)^37–41^. In our model, from a pathogen’s perspective, sex can be thought as a two-state heterogeneous environment. Consistent with previous work, we find that epigenetic cues may evolve when they provide information on the sex of the current host, and this information cannot be obtained directly—for example, due to a delay between the moment of infection and the time when information on the sex of the host may be available. In addition, we find that epigenetic cues may evolve when they provide information on attributes of the population in which the host is found. In particular, such cues are of value when information on the sex of the past host informs about future transmission opportunities.

To support the evolution of origin-specific and/or origin-&-sex-specific virulence there must be differences between either host sexes in their infectivity and susceptibility, or a population structured according to differences in the interactions between the sexes. These biological and social differences are pervasive in human populations: differences between the sexes in infectivity and in susceptibility are ubiquitous^42–48^; differences between social groups with respect to their sex ratios and/or the contact patterns between individuals of the same and opposite sexes have been widely reported^49,50^. The outcome will be virulence that differs when the pathogen was transmitted from a female rather than a male (origin-specific virulence) or virulence that differs when the pathogen was transmitted from a female and infecting a female, or transmitted from a male and infecting a female (origin-&-sex-specific virulence). The latter complex patterns of virulence can result in greater average virulence in same-sex infections than cross-sex transmission infections or vice versa.

**Fig. 10:**
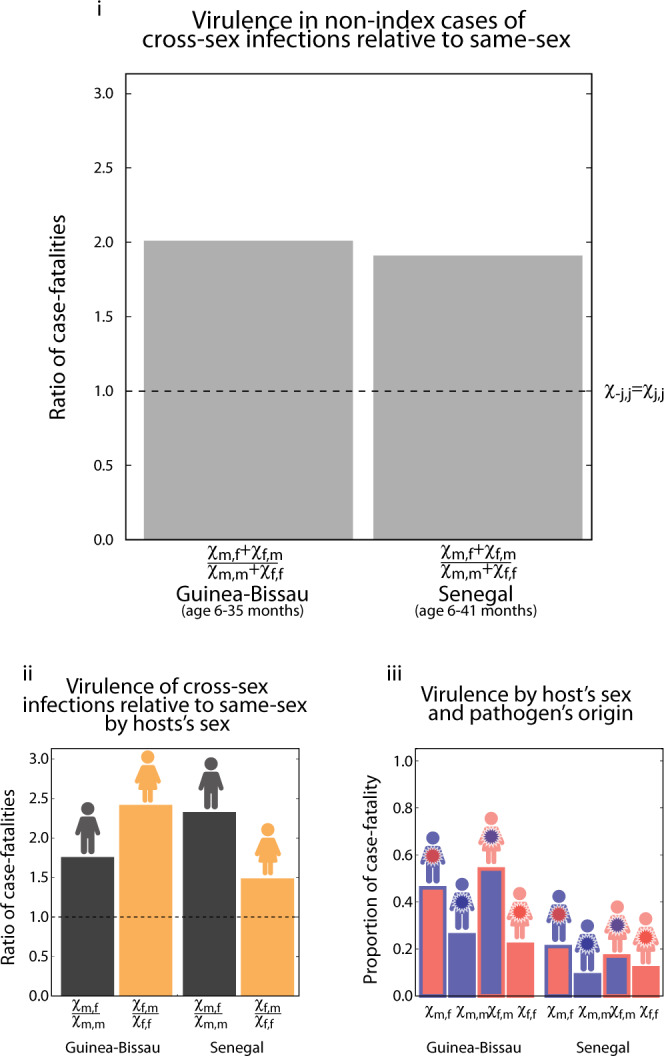
Virulence of cross-sex and same-sex measles infections. Sub-panel **i** shows the ratio of case fatalities (*χ*) in cross-sex infections relative to same-sex infections, defined as (*χ*_*mf*_ + *χ*_*fm*_)/(*χ*_*mm*_ + *χ*_*ff*_), in two studies conducted in children 6-35 months old in Guinea-Bissau and Senegal. Notice that in both cases infections from the opposite sex are roughly twice as virulent as infections from the same sex. Sub-panel **ii** shows the ratio of case fatalities in cross-sex infections relative to same-sex infections broken down by sex of the infected. This ratio in infected boys is given by the proportion of boys infected by girls relative to the one infected by boys, that is *χ*_*mf*_/*χ*_*mm*_ and in infected girls is given by the proportion of girls infected by boys relative to the one infected by girls, that is *χ*_*fm*_/*χ*_*ff*_. Notice that in all cases infections from the opposite-sex are more virulent than infections from the same-sex, that is *χ*_¬*jj*_/*χ*_*jj*_ > 1, ranging between 1.5 and 2.5 times more virulent. Finally, sub-panel **iii** shows the proportion of case fatalities in each of the sexes when infected by each of the other sexes in the two studies conducted in Guinea-Bissau and Senegal^23,24^.

Our findings offer an explanation for the complex pattern of virulence identified by doctors in diseases like measles, chickenpox and polio. More than two decades ago, it was observed that in countries with limited access to medical treatment, girls infected with measles by boys were 2.5 times more likely to die from the infection than girls infected by other girls^23^ (Fig. 10i). Similarly, boys infected by girls were 1.7 times more likely to die than boys infected by other boys (Fig. 10ii). Overall, average cross-sex infections with measles were twice as virulent when compared to average same-sex infections (Fig. 10iii). This same qualitative result has been replicated by other studies^24^ and extended to infections with chickenpox and polio^25,26^. While these complex patterns of virulence are well established empirically, their origins remain a mystery^27^. Focusing on the host, differences in virulence between cross-sex and same-sex infections cannot be explained by sex differences in the immune system or in gene expression alone. The latter would result in different virulence in girls and boys but the lack of effects due to the origin of the infection would result in the cancellation of these differences; if there were only sex-specific effects caused by the immune system, cross-sex and same-sex virulence would not differ on average.

We argue that epigenetically inherited and acquired marks allow the evolution of sex-&-origin-specific virulence in pathogens in ways that can explain greater virulence of cross-sex infections as opposed to same-sex infections, as observed in measles, chickenpox and polio^23–26^. We devised two plausible scenarios in which such complex patterns may evolve; both cases require a population structure where subpopulations differ with respect to their contact patterns in a sex-specific manner. For example, a population where the pathogen is transmitted within two groups of individuals. One group may be formed largely by individuals with primarily same-sex interactions, and the other group by individuals with largely cross-sex interactions. One way in which greater virulence of cross-sex infections may evolve is when, in addition to the aforementioned contact structure, the two groups differ in their sex ratio and/or size (Fig. 6). Alternatively, greater virulence in cross-sex infections may evolve when in addition to the aforementioned contact structure there is greater same-sex transmissibility (Fig. 7).

**Fig. 11:**
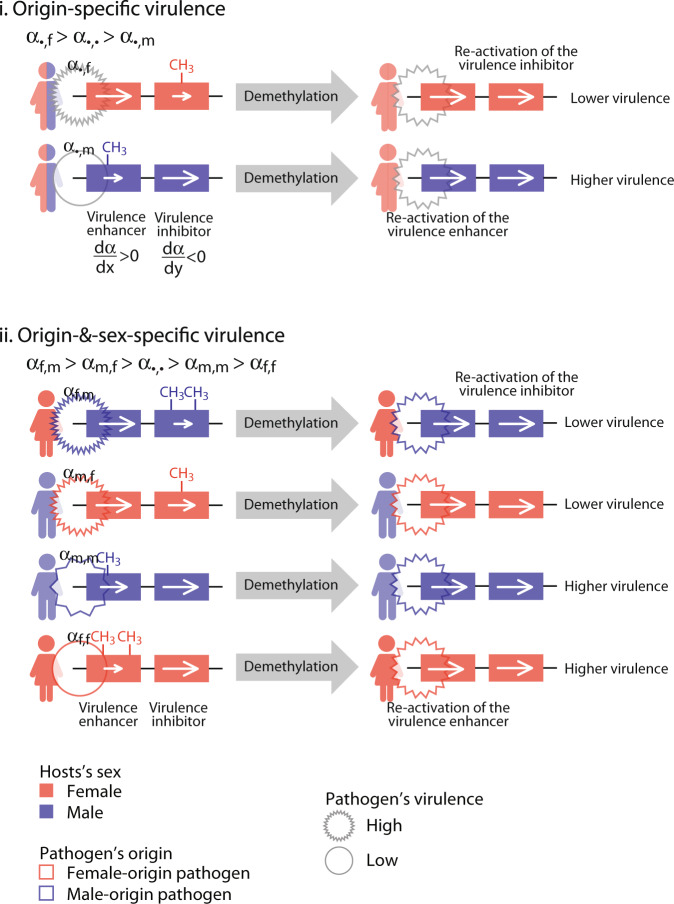
Predictions for the effects of epigenetic therapy on the virulence of an infection. Here we consider haploid pathogens with two types of genes: one whose greater expression *x* enhances virulence, $\frac{d\alpha }{dx}\; > \; 0$, (henceforth *virulence enhancer*), and another one whose greater expression *y* inhibits virulence, $\frac{d\alpha }{dy}\;<\; 0$, (henceforth *virulence inhibitor*). Epigenetically inherited marks are established by methylation of promoter regions resulting in reduced expression of the gene. Epigenetic interventions consist of demethylating the pathogen’s genome. In sub-panel **i**, we consider pathogens exhibiting origin-specific virulence. When infections originating in females are more virulent (*α*_•*f*_ > *α*_•*m*_), pathogens of female-origin are selected to methylate the promoter of virulence inhibitor genes thus increasing the virulence of the infection. Therefore epigenetic interventions that demethylate infections from females will re-activate virulence inhibitors and reduce the virulence of the infection. In sub-panel **ii**, we consider pathogens showing origin-&-sex-specific virulence. When the virulence of pathogens with male-origin in females is higher (*α*_*fm*_ > *α*_*mf*_ > *α*_*mm*_ > *α*_*ff*_), pathogens with male-origin in females are selected to methylate the promoter of virulence inhibitor genes thus increasing the virulence of the infection. Thus epigenetic interventions that demethylate infections in females originating from females, will re-activate virulence inhibitors and thus reduce the virulence of the infection. Notice, however, that pathogens with the same male-origin but infecting females, will respond to the same epigenetic intervention in the opposite manner, that is increasing the virulence of the infection.

Our research suggests the possibility of developing epigenetic therapies by interfering with pathogens’ memories. Epigenetic therapies have been widely used in the treatment of cancer^9,51^, and their use in the treatment of infectious diseases is starting to be implemented. If evidence of epigenetic marks modifying the virulence of pathogens transmitted from different sexes were to be found, it would be possible to use the predictions of our model to guide modifications of the epigenome of pathogens exhibiting origin-specific virulence. When pathogens of male origin are selected for lower virulence they will under-express any gene whose expression is positively correlated with virulence (‘virulence enhancer’) (Fig. 11). By contrast, when pathogens of female origin are selected for higher virulence, they will underexpress any gene whose expression is negatively correlated with virulence (‘virulence inhibitor’) (Fig. 11).

Epigenetic marks in viruses and bacteria are often implemented by downregulating the expression of genes via differential methylation of the promoter of a gene^3,52^, although this is not the only mechanism and the mechanisms are more diverse in other pathogens^53,54^. While our model applies to any mechanism of non-genetic inheritance, for the purpose of illustrating how our model may guide epigenetic therapy we focus on marks established via methylation of the promoter. In particular, here we explore an epigenetic intervention to reduce the virulence of an infection by demethylating the pathogen’s genome using, as a criterion, the sex of the host from which the pathogen originated. This can be achieved through different pharmacological agents, i.e generic DNA methyltransferase inhibitors^53,54^ or specific use of CRISPR to demethylate a gene^55^. When natural selection results in higher virulence of infections originating from a given sex, we predict that genome-wide demethylation of the pathogen originated from that sex, and only that sex, will reduce virulence (Fig. 11). This is because demethylation will upregulate previously muted virulence inhibitors in pathogens originating from one sex, but will upregulate previously muted virulence enhancers in pathogens originating from the other sex (Fig. 11). Importantly, indiscriminate demethylation will produce undesirable effects when directed at pathogens without accounting for their origin. For specific patterns of virulence, our model allows us to predict the outcome of treatments that demethylate the pathogen’s genome.

More generally, our work suggests that complex patterns of virulence (origin-specific or origin-&-sex specific) can be understood in terms of the selective pressures that affect the pathogen. We propose the intriguing possibility that pathogen’s memories of past environments may be driving the virulence of infections. Our research focuses on the sex of the previous host but our results could be extended to other characters (e.g., stress levels caused by differences in the socioeconomic status of hosts). The possibility that there may be a variety of epigenetically inherited memories advocates the need for personalised approaches to infections in which factors like sex or social status can inform the best course of action to treat a disease. This research aims to be a first step in that direction.

## Methods

We identify Evolutionarily Stable levels of virulence using a numerical invasion analysis implemented in Matlab^56^. A detailed description of our approach can be found in Note 2.1 of the Supplementary Information, and custom code and the numerical data it generates are available for download. Briefly, results are obtained by guessing the ES levels of virulence, then carrying out the following five-step procedure: (i) Substitute the most recent guess into the system of equations in (1), then integrate forward in time to find the equilibrium levels of susceptible and infected hosts. (ii) Use the equilibrium levels of susceptible and infected hosts to construct the selection gradients described in (2). (iii) Update the guess by either incrementing it (when the corresponding selection gradient is positive) or decrementing it (when the corresponding selection gradient is negative). (iv) Repeat (i-iii) until the magnitude of each relevant selection gradient is within a tolerance of zero. (v) Return each of the most recent guesses as the estimates of the various ES virulence levels.

### Reporting summary

Further information on research design is available in the Nature Research Reporting Summary linked to this article.

## Supplementary information

Supplementary Information

Reporting Summary

## Data Availability

The numerical results used to produce Figs 4–7 and figures that appear in the Supplementary Information can be found at https://github.com/geoffwild/McLeod_et_al_NComm and at https://zenodo.org/deposit/4939424.

## Code availability

Custom code was written using Matlab^56^ and can be found at https://github.com/geoffwild/McLeod_et_al_NComm and at https://zenodo.org/deposit/4939424.
