# Supplementary material for: Epigenetic memories and the evolution of infectious diseases
Source: Nat Commun. 2021 Jul 13;12:4273. doi: 10.1038/s41467-021-24580-0 (PMC8277771; doi:10.1038/s41467-021-24580-0)
Supplement: Supplementary file 1 — Supplementary Information [file 41467_2021_24580_MOESM1_ESM.pdf]

Supplementary Information for: Epigenetic memories and the evolution of infectious diseases

David V. McLeod[1,2], Geoff Wild[3] and Francisco Úbeda[4†]

[1] Centre D'Ecologie Fonctionnelle & Evolutive, CNRS

34090 Montpellier, France

[2] Institute of Integrative Biology, ETH Zürich

8092 Zürich, Switzerland

[3]Department of Applied Mathematics, The University of Western Ontario

London, Ontario N6A 5B7, Canada

[4]Department of Biology, Royal Holloway University of London

Egham, Surrey TW20 0EX, United Kingdom

[†]To whom correspondence should be addressed; E-mail: f.ubeda@rhul.ac.uk

Note 1 Model

Consider a population of two types of hosts, $i = 1, 2$, subdivided into two subpopulations, $\ell = A, B$. We will assume host type corresponds to host sex. Let S_i^ℓ denote the density of susceptible i-sex hosts in subpopulation ℓ, and let $x_{j,k}^\ell$ denote the density of infected j-sex hosts in subpopulation ℓ who were infected by a k-sex host. Hosts move from one subpopulation to the other at a per-capita rate σ either through dispersal (if the subpopulations represent spatial subdivision) or behavioural change (if the subpopulations are associated with different contact networks).

Infection is transmitted from an infected host of type (j, k) to a susceptible host of type i according to a law of mass action with rate constant $\beta_{i \leftarrow (j,k)}$, so the transmission chain is $k \to j \to i$. A pathogen of infection type (j, k) imposes disease-related mortality (virulence) on its host at per-capita rate $\alpha_{j,k}$, while hosts experience natural mortality and recovery (without immunity) at rates μ and γ, respectively. Although the subpopulations could differ with respect to a variety of aspects, we limit our attention to two possible differences: differences in host contact networks and differences in sex ratio. An example of the former is a sexually-transmitted infection with a population subdivided into heterosexual and homosexual contact networks, whereas an example of the latter is a population in which individuals tend to be grouped by sex. We let $c_{i \leftrightarrow j}^\ell$ control the probability of contact between a host of sex i and sex j in subpopulation ℓ, such that $c_{i \leftrightarrow j}^\ell = c_{j \leftrightarrow i}^\ell$, and we let Λ_i^ℓ denote the influx of new susceptibles of sex i to subpopulation ℓ such that $\sum_i \Lambda_i^\ell = \Lambda$.

Under these assumptions the dynamics of the host population can be captured by

the following system of eight differential equations (see also Fig. 1):

$$\begin{aligned}
\dot{S}_i^A &= \Lambda_i^A - \mu S_i^A - S_i^A \sum_j c_{i\leftrightarrow j}^A \sum_k \beta_{i\leftarrow(j,k)} x_{j,k}^A + \gamma \sum_j x_{i,j}^A + \sigma S_i^B - \sigma S_i^A \\
\dot{S}_i^B &= \Lambda_i^B - \mu S_i^B - S_i^B \sum_j c_{i\leftrightarrow j}^B \sum_k \beta_{i\leftarrow(j,k)} x_{j,k}^B + \gamma \sum_j x_{i,j}^B + \sigma S_i^A - \sigma S_i^B \\
\dot{x}_{j,k}^A &= S_j^A c_{j\leftrightarrow k}^A \sum_i \beta_{j\leftarrow(k,i)} x_{k,i}^A - (\alpha_{j,k} + \mu + \gamma) x_{j,k}^A + \sigma x_{j,k}^B - \sigma x_{j,k}^A \\
\dot{x}_{j,k}^B &= S_j^B c_{j\leftrightarrow k}^B \sum_i \beta_{j\leftarrow(k,i)} x_{k,i}^B - (\alpha_{j,k} + \mu + \gamma) x_{j,k}^B + \sigma x_{j,k}^A - \sigma x_{j,k}^B.
\end{aligned} \tag{1}$$

We make the standard assumption that transmission is an increasing function of virulence, that is, $\beta_{i\leftarrow(j,k)} = \beta_{i\leftarrow(j,k)}(\alpha_{j,k})$ and $\frac{\mathrm{d}\beta_{i\leftarrow(j,k)}}{\mathrm{d}\alpha_{j,k}} > 0$ and so we will use the function

$$\beta_{i\leftarrow(j,k)}(\alpha) = \frac{\alpha}{\alpha + \theta_{i\leftarrow(j,k)}} \tag{2}$$

to model the relationship between transmission and disease-related mortality.

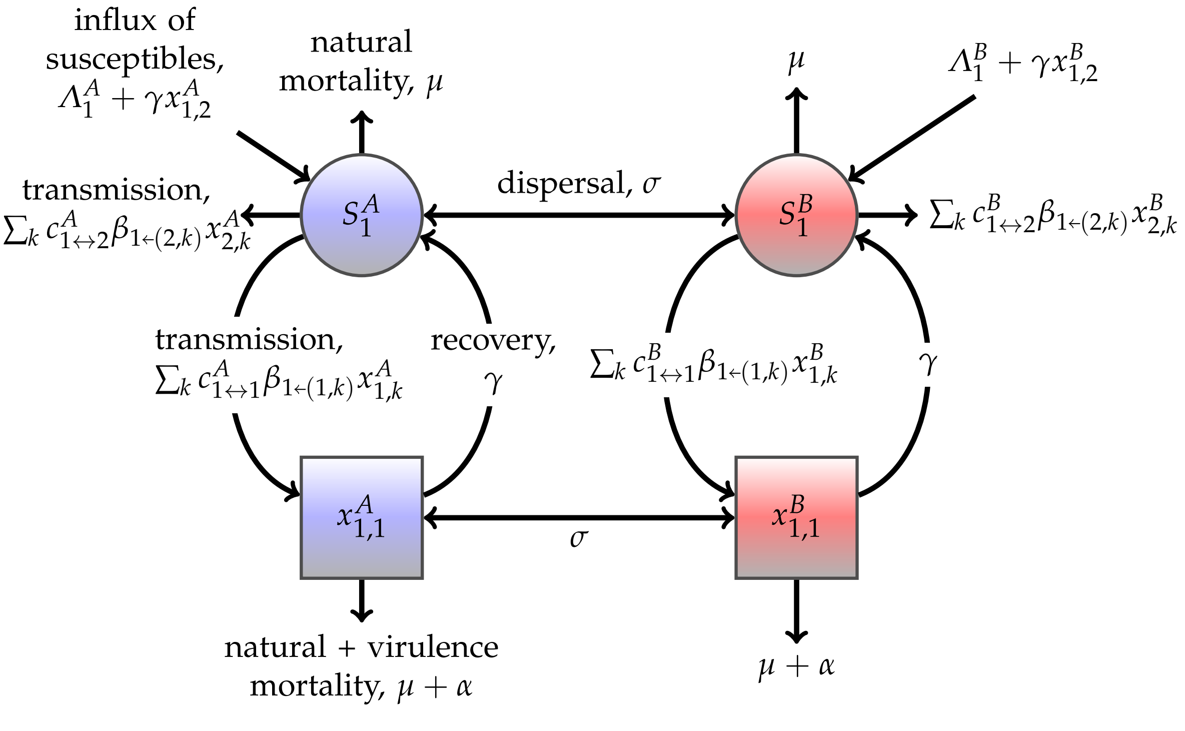

Supplementary Figure 1: Flow diagram of the population dynamics captured by (1). For visual clarity, we have only included one susceptible host sex, sex 1, and one class of infected hosts, $(1, 1)$; the other classes and sexes of hosts follow a similar pattern.

Note 2 General analysis

Our interest is the evolution of the virulence traits $\alpha_{j,k}$. As such, we will focus upon invasion analyses and finding the 'evolutionarily stable' (ES) virulence. To do so, let $\tilde{\alpha}_{j,k}$ be the mutant version of the resident trait $\alpha_{j,k}$, and let $\boldsymbol{\alpha} = [\alpha_{1,1}, \alpha_{1,2}, \alpha_{2,1}, \alpha_{2,2}]$ be the vector of resident virulence traits (and $\tilde{\boldsymbol{\alpha}}$ its mutant counterpart). Then using an invasion analysis[1], the success of the rare mutant strain depends on the eigenvalues of the 8×8 matrix

$$\mathbf{A}(\tilde{\boldsymbol{\alpha}}) = \begin{bmatrix} \mathbf{F}^A(\tilde{\boldsymbol{\alpha}}) - \mathbf{V}(\tilde{\boldsymbol{\alpha}}) & \sigma\mathbf{I} \\ \sigma\mathbf{I} & \mathbf{F}^B(\tilde{\boldsymbol{\alpha}}) - \mathbf{V}(\tilde{\boldsymbol{\alpha}}) \end{bmatrix}.$$

Here, $\mathbf{I}$ is the 4×4 identity matrix, and the 4×4 matrices $\mathbf{F}^\ell(\tilde{\boldsymbol{\alpha}})$ and $\mathbf{V}(\tilde{\boldsymbol{\alpha}})$ are given by

$$\mathbf{F}^\ell(\tilde{\boldsymbol{\alpha}}) = \begin{bmatrix} \bar{S}_1^\ell c_{1\leftrightarrow 1}^\ell \beta_{1\leftarrow(1,1)}(\tilde{\alpha}_{1,1}) & \bar{S}_1^\ell c_{1\leftrightarrow 1}^\ell \beta_{1\leftarrow(1,2)}(\tilde{\alpha}_{1,2}) & 0 & 0 \\ 0 & 0 & \bar{S}_1^\ell c_{1\leftrightarrow 2}^\ell \beta_{1\leftarrow(2,1)}(\tilde{\alpha}_{2,1}) & \bar{S}_1^\ell c_{1\leftrightarrow 2}^\ell \beta_{1\leftarrow(2,2)}(\tilde{\alpha}_{2,2}) \\ \bar{S}_2^\ell c_{1\leftrightarrow 2}^\ell \beta_{2\leftarrow(1,1)}(\tilde{\alpha}_{1,1}) & \bar{S}_2^\ell c_{1\leftrightarrow 2}^\ell \beta_{2\leftarrow(1,2)}(\tilde{\alpha}_{1,2}) & 0 & 0 \\ 0 & 0 & \bar{S}_2^\ell c_{2\leftrightarrow 2}^\ell \beta_{2\leftarrow(2,1)}(\tilde{\alpha}_{2,1}) & \bar{S}_2^\ell c_{2\leftrightarrow 2}^\ell \beta_{2\leftarrow(2,2)}(\tilde{\alpha}_{2,2}) \end{bmatrix}$$

where the overbar notation, $\bar{S}_i^\ell$, indicates the equilibrium density of susceptibles of type i in the resident subpopulation ℓ, and

$$\mathbf{V}(\tilde{\boldsymbol{\alpha}}) = \begin{bmatrix} \tilde{\alpha}_{1,1} + \mu + \gamma & & & \\ & \tilde{\alpha}_{1,2} + \mu + \gamma & & \\ & & \tilde{\alpha}_{2,1} + \mu + \gamma & \\ & & & \tilde{\alpha}_{2,2} + \mu + \gamma \end{bmatrix}.$$

The dominant eigenvalue of $\mathbf{A}(\boldsymbol{\alpha})$ is zero, and its associated right eigenvector, $\mathbf{w}$, is proportional to the equilibrium population densities of the different classes of individuals

infected with the resident strain,

$$\mathbf{w}^T = [\bar{x}_{1,1}^A,\ \bar{x}_{1,2}^A,\ \bar{x}_{2,1}^A,\ \bar{x}_{2,2}^A,\ \bar{x}_{1,1}^B,\ \bar{x}_{1,2}^B,\ \bar{x}_{2,1}^B,\ \bar{x}_{2,2}^B].$$

Similarly, the left eigenvector, $\mathbf{v}$, associated with the zero eigenvalue is proportional to the reproductive values of the corresponding individual infections,

$$\mathbf{v} = [v_{1,1}^A,\ v_{1,2}^A,\ v_{2,1}^A,\ v_{2,2}^A,\ v_{1,1}^B,\ v_{1,2}^B,\ v_{2,1}^B,\ v_{2,2}^B].$$

Using standard techniques[2], the gradient of selection acting on trait $\alpha_{j,k}$ at $\alpha_{j,k}$ is

$$\lambda'_{j,k}(\alpha_{j,k}) \equiv \mathbf{v}^T \frac{\partial \mathbf{A}}{\partial \alpha_{j,k}} \mathbf{w} = \sum_{\ell} \underbrace{\left[\sum_{m} v_{m,j}^{\ell} c_{m\leftrightarrow j}^{\ell} \frac{\mathrm{d}\beta_{m\leftarrow(j,k)}}{\mathrm{d}\alpha_{j,k}} \bar{S}_m^{\ell} - v_{j,k}^{\ell} \right]}_{\frac{\mathrm{d}\mathcal{R}_{j,k}^{\ell}}{\mathrm{d}\alpha_{j,k}}} \bar{x}_{j,k}^{\ell}. \tag{3}$$

The gradient of selection given by (3) has a standard interpretation. In particular, $\mathcal{R}_{j,k}$ is the production of new infections, weighted by the reproductive value of the type of infectious individual created (the $v_{m,j}^{\ell}$) minus the mortality rate of an individual in class (j,k) (i.e., $\mu + \gamma + \alpha_{j,k}$), weighted by the reproductive value, $v_{j,k}^{\ell}$. Thus the derivative with respect to $\alpha_{j,k}$ represents the effect a one unit change in virulence has upon the fitness of a (j,k) infection. This is summed across subpopulations since we do not allow for the possibility of conditional virulence expression based upon subpopulation: this information is "hidden" from the pathogen.

If we are interested in one population alone, we will focus exclusively upon the set of equations indexed by either the superscript A or B and set $\sigma = 0$ and $c_{i\leftrightarrow j}^{\ell} = 1$, since for one subpopulation the $c_{i\leftrightarrow j}^{\ell}$ can be subsumed into the $\beta_{i\leftarrow(j,k)}(\alpha)$. Then from (3) the

gradient of selection acting on $\alpha_{j,k}$ is

$$\lambda'_{j,k}(\alpha_{j,k}) = \left(\sum_m v_{m,j} \frac{\mathrm{d}\beta_{m \leftarrow (j,k)}}{\mathrm{d}\alpha_{j,k}} \bar{S}_m - v_{j,k} \right) \bar{x}_{j,k}, \tag{4}$$

where we have dropped the superscript ℓ.

To indicate specific cases in which a quantity, originally subscripted with each of i, j, and k is to be considered as being independent of the factors corresponding to a given index we will use a $\bullet$. For example, $\beta_{\bullet \leftarrow (j,k)}$ means $\beta_{i \leftarrow (j,k)}$ depends upon j and k but not i, so $\beta_{1 \leftarrow (j,k)} = \beta_{2 \leftarrow (j,k)}$. Using this notation, from (3) the gradient of selection for the evolution of virulence in the absence of information regarding origin of the pathogen and sex of the host, $\alpha_{\bullet,\bullet}$, is

$$\lambda'(\alpha_{\bullet \leftarrow \bullet}) \equiv \sum_j \sum_k \lambda'_{j \leftarrow k}(\alpha_{\bullet,\bullet}). \tag{5}$$

The gradient of selection for the evolution of virulence conditional on the origin of the pathogen (henceforth 'origin-specific virulence') $\alpha_{\bullet,k}$ is

$$\lambda'_{\mathrm{O}(k)}(\alpha_{\bullet,k}) \equiv \sum_j \lambda'_{j,k}(\alpha_{\bullet,k}), \tag{6}$$

while the gradient of selection for the evolution of virulence conditional on the sex of the pathogen's host (henceforth 'sex-specific virulence') $\alpha_{j,\bullet}$ is

$$\lambda'_{\mathrm{S}(j)}(\alpha_{j,\bullet}) \equiv \sum_k \lambda'_{j,k}(\alpha_{j,\bullet}). \tag{7}$$

With these definitions in mind, equation (3) is the gradient of selection for the evolution of virulence conditional on the origin of the pathogen and sex of the pathogen's host (henceforth 'origin-and-sex-specific virulence') $\alpha_{j,k}$. The level of virulence at which the gradient of selection is zero is a candidate ES virulence.

Our objective is to understand how virulence evolves subject to different constraints and so we consider each of sex-specific, origin-specific and origin-and-sex-specific virulence separately. For each case, we seek to address two questions:

(1) When can a pathogen displaying either sex-specific, origin-specific, or origin-and-sex-specific virulence invade a population in which the resident virulence trait is not plastic, $\alpha_{\bullet,\bullet}$?

(2) Given either sex- or origin-specific virulence invades, what factors are most likely to cause one of the conditional ES virulences, that is, $\alpha_{\bullet,1}$ or $\alpha_{1,\bullet}$, be expressed at a level that is greater than the other, that is, either $\alpha_{\bullet,1} > \alpha_{\bullet,2}$ or $\alpha_{1,\bullet} > \alpha_{2,\bullet}$? And, given origin-and-sex-specific virulence invades, under what circumstances is cross-sex transmission likely to lead to greater ES virulence than same-sex transmission?

In answering these questions we consider both the one-population and two subpopulation models. For reference, in the one population model, the left eigenvector can be written

$$\mathbf{v} = \left[\left(\frac{\alpha_{1,2} + \mu + \gamma}{\alpha_{2,1} + \mu + \gamma} \right) \frac{\beta_{2\leftarrow(1,1)}}{\beta_{1\leftarrow(1,2)}} \frac{\bar{S}_2}{\bar{S}_1} \bar{x}_{1,1},\ \bar{x}_{2,1},\ \left(\frac{\alpha_{1,2} + \mu + \gamma}{\alpha_{2,1} + \mu + \gamma} \right) \bar{x}_{1,2},\ \frac{\beta_{1\leftarrow(2,2)}}{\beta_{2\leftarrow(2,1)}} \frac{\bar{S}_1}{\bar{S}_2} \bar{x}_{2,2} \right]. \tag{8}$$

Note 2.1 Numerical analysis overview

In some cases, the complexity of (1) means that we must rely upon numeric calculations to find the ES virulence. All numerical calculations were performed in Matlab[3] and the code is available through github and zenodo (see Code Availability Section of the main text). When we use numerical calculations, we follow the same procedure in all cases. As an example, suppose we want to find the ES origin-and-sex-specific virulence for a given parameter set, starting from some initial virulence value, say $\alpha_{j,k}^{\text{old}}$. Then

(1) Using $\alpha_{j,k}^{\text{old}}$, we integrate the ODE system (1) until

$$\sqrt{\sum_{i,\ell}(\dot{S}_i^\ell)^2 + \sum_{j,k,\ell}(\dot{x}_{j,k}^\ell)^2} < \epsilon_0$$

where ϵ_0 is some pre-specified value. If t_n is the time at which this occurs, the solution values at t_n are our demographic equilibria, e.g., $\bar{x}_{1,1}^A = x_{1,1}^A(t_n)$.

(2) Using these demographic equilibria, we compute the selection gradient for the case of interest, either (3) (for 2 subpopulations) or (4) (for one population).

(3) We then compute the new value of $\alpha_{j,k}$ for the next iteration as

$$\alpha_{j,k}^{\text{new}} = \alpha_{j,k}^{\text{old}} + \Delta\alpha\lambda'_{j,k}(\alpha_{j,k}^{\text{old}})$$

where $\Delta\alpha$ is some pre-specified baseline mutation step which is then scaled by the magnitude (and sign) of the gradient of selection.

(4) We then set $\alpha_{j,k}^{\text{old}} = \alpha_{j,k}^{\text{new}}$, and repeat this process until the norm of the gradients of selection is below a prespecified value, ϵ_1,

$$\sqrt{\sum_{j,k}(\lambda'_{j,k}(\alpha_{j,k}^{\text{old}}))^2} < \epsilon_1.$$

The values $\alpha_{j,k}^{\text{old}}$ satisfying this are taken to be the ES virulence.

In general, when we are considering the evolution of virulence plasticity, we first find the fully-constrained ES virulence, $\alpha_{\bullet,\bullet}^*$ for a given parameter set, and then use this as the initial virulence value, i.e., $\alpha_{j,k}^{\text{old}} = \alpha_{\bullet,\bullet}^*$.

Note 3 Sex-specific virulence

We include results for the evolution of sex-specific virulence for reference.

Note 3.1 When does sex-specific virulence invade?

First consider the one population model. It is simpler to state when sex-specific virulence will not invade: only when transmission does not depend upon current host, $\beta_{i\leftarrow(\bullet,k)}(\alpha)$ (note that $\beta_{i\leftarrow(\bullet,k)}(\alpha)$ includes the possibility of both $\beta_{i\leftarrow(\bullet,\bullet)}(\alpha)$ and $\beta_{\bullet\leftarrow(\bullet,k)}(\alpha)$).

To see this, note that at demographic equilibrium, $\bar{x}_{1,2} = \bar{x}_{2,1}$ and $\bar{x}_{1,2}\bar{x}_{2,1} = \bar{x}_{1,1}\bar{x}_{2,2}$; this can be obtained from (1). Using these relations in (8) gives

$$\mathbf{v} = \left[v_{1,1},\, v_{1,2},\, v_{1,2},\, \frac{(v_{1,2})^2}{v_{1,1}} \right]. \tag{9}$$

As a result, we can write

$$\frac{\mathrm{d}\mathcal{R}_{2,1}}{\mathrm{d}\alpha} = \frac{v_{1,2}}{v_{1,1}} \frac{\mathrm{d}\mathcal{R}_{1,1}}{\mathrm{d}\alpha} \quad \text{and} \quad \frac{\mathrm{d}\mathcal{R}_{2,2}}{\mathrm{d}\alpha} = \frac{v_{1,2}}{v_{1,1}} \frac{\mathrm{d}\mathcal{R}_{1,2}}{\mathrm{d}\alpha}. \tag{10}$$

Using these relations in (6) gives

$$\lambda'_{S(1)}(\alpha_{\bullet,\bullet}) = \frac{v_{1,2}}{v_{1,1}} \frac{\bar{x}_{1,1}}{\bar{x}_{1,2}} \lambda'_{S(2)}(\alpha_{\bullet,\bullet}),$$

and so sex-specific virulence will not invade. If instead transmission does depend upon sex of current host, sex-specific virulence will invade.

In the two subpopulation model, provided there are differences between subpopulations either in terms of contact networks $c^{\ell}_{i\leftrightarrow j}$, or influx of hosts, $\Lambda_i^A \neq \Lambda_i^B$, sex-specific virulence will invade even when it cannot in the one population model (i.e., $\beta_{i\leftarrow(\bullet,k)}(\alpha)$). The reason why this occurs is simple: sex of the current host provides some information about the subpopulation the pathogen is currently in.

Note 3.2 What factors lead to greater virulence in one sex?

Again, focus upon the one population model. Given sex-specific virulence invades, will pathogens be more virulent in hosts of sex 1 or 2? All else being equal whichever

sex (say 1) has lower transmissibility will experience greater sex-specific virulence, that is, if $\beta_{i\leftarrow(1,k)}(\alpha) < \beta_{i\leftarrow(2,k)}(\alpha)$ holds for all i and k, then $\alpha_{1,\bullet} > \alpha_{2,\bullet}$. This is a standard prediction of transmission-virulence trade-off theory: reducing transmission selects for greater virulence see e.g.,[4,5].

Note 4 Origin-specific virulence

Note 4.1 One population

Note 4 When does origin-specific virulence invade?

As before it is easier to state the conditions under which origin-specific virulence cannot invade. In particular, it will not invade if either transmission depends only upon sex of current host, $\beta_{\bullet\leftarrow(j,\bullet)}(\alpha)$ or transmission depends only upon sex of the future host, $\beta_{i\leftarrow(\bullet,\bullet)}(\alpha)$. This can be reasoned as follows. First, clearly if transmission depends directly upon host-of-origin, then origin-specific virulence will invade. Therefore suppose not, that is, $\beta_{i\leftarrow(j,\bullet)}(\alpha)$. In this circumstance, the left eigenvector takes the form of

$$\mathbf{v} = [v_{1,1}, v_{1,1}, v_{2,1}, v_{2,2}], \tag{11}$$

and so as a result

$$\frac{d\mathcal{R}_{1,1}}{d\alpha} = \frac{d\mathcal{R}_{1,2}}{d\alpha} = \frac{d\mathcal{R}_{1,\bullet}}{d\alpha} \quad \text{and} \quad \frac{d\mathcal{R}_{2,1}}{d\alpha} = \frac{d\mathcal{R}_{2,2}}{d\alpha} = \frac{d\mathcal{R}_{2,\bullet}}{d\alpha}. \tag{12}$$

At the no plasticity ES virulence, $\alpha^*_{\bullet,\bullet}$, we must have $\sum_j \sum_k \lambda'_{j,k}(\alpha^*_{\bullet,\bullet}) = 0$ and so it follows that $\lambda'_{O(1)}(\alpha^*_{\bullet,\bullet}) = -\lambda'_{O(2)}(\alpha^*_{\bullet,\bullet})$. Therefore for origin-specific virulence to *not* invade, it suffices to show that $\lambda'_{O(1)}(\alpha^*_{\bullet,\bullet}) = 0$, that is, $\alpha^*_{\bullet,\bullet}$ is also the origin-specific ES virulence. We do this now.

Suppose we are at the ES virulence for no plasticity, $\alpha^*_{\bullet,\bullet}$. Then

$$\lambda'(\alpha^*_{\bullet,\bullet}) = \left[\frac{\mathrm{d}\mathcal{R}_{1,\bullet}}{\mathrm{d}\alpha}(\bar{x}_{1,1}+\bar{x}_{1,2}) + \frac{\mathrm{d}\mathcal{R}_{2,\bullet}}{\mathrm{d}\alpha}(\bar{x}_{2,1}+\bar{x}_{2,2})\right]_{\alpha=\alpha^*_{\bullet,\bullet}} = 0, \tag{13}$$

where we have used the relations (11) and (12) and so it follows that

$$\left[\frac{\mathrm{d}\mathcal{R}_{1,\bullet}}{\mathrm{d}\alpha}\Big/\frac{\mathrm{d}\mathcal{R}_{2,\bullet}}{\mathrm{d}\alpha}\right]_{\alpha=\alpha^*_{\bullet,\bullet}} = -\frac{\bar{x}_{2,1}+\bar{x}_{2,2}}{\bar{x}_{1,1}+\bar{x}_{1,2}}.$$

Using this information in (6) reveals that

$$\lambda'_{\mathrm{O}(1)}(\alpha^*_{\bullet,\bullet}) = (\bar{x}_{2,1}+\bar{x}_{2,2})\left(\frac{\bar{x}_{2,1}}{\bar{x}_{2,1}+\bar{x}_{2,2}} - \frac{\bar{x}_{1,1}}{\bar{x}_{1,1}+\bar{x}_{1,2}}\right)\frac{\mathrm{d}\mathcal{R}_{2,\bullet}}{\mathrm{d}\alpha}\bigg|_{\alpha=\alpha^*_{\bullet,\bullet}} \tag{14}$$

So if either $\frac{\bar{x}_{2,1}}{\bar{x}_{2,1}+\bar{x}_{2,2}} = \frac{\bar{x}_{1,1}}{\bar{x}_{1,1}+\bar{x}_{1,2}}$ or $\frac{\mathrm{d}\mathcal{R}_{2,\bullet}}{\mathrm{d}\alpha} = 0$, origin-specific virulence will not invade. Using a computer algebra package (e.g. we used the Maple[6] package), it can be shown that

$$\frac{\bar{x}_{2,1}}{\bar{x}_{2,1}+\bar{x}_{2,2}} - \frac{\bar{x}_{1,1}}{\bar{x}_{1,1}+\bar{x}_{1,2}} \propto \beta_{2\leftarrow(1,\bullet)}\beta_{1\leftarrow(2,\bullet)} - \beta_{1\leftarrow(1,\bullet)}\beta_{2\leftarrow(2,\bullet)}. \tag{15}$$

By inspection we see that if $\beta_{i\leftarrow(\bullet,\bullet)}$ or $\beta_{\bullet\leftarrow(j,\bullet)}$, then from (15) $\frac{\bar{x}_{2,1}}{\bar{x}_{2,1}+\bar{x}_{2,2}} = \frac{\bar{x}_{1,1}}{\bar{x}_{1,1}+\bar{x}_{1,2}}$ and so origin-specific virulence cannot invade. In Figure 2 we show that for all other cases, origin-specific virulence can invade.

Note 4 What factors lead to greater origin-specific virulence?

Given origin-specific virulence invades, will pathogens originating from hosts of sex 1 or 2 be more virulent? There are two cases of interest: when transmission depends upon host-of-origin, and when it does not. We consider each in turn.

(1) Transmission depends upon host-of-origin. Then if we have

$$\beta_{\bullet\leftarrow(\bullet,2)}(\alpha) \geq \beta_{\bullet\leftarrow(\bullet,1)}(\alpha)$$

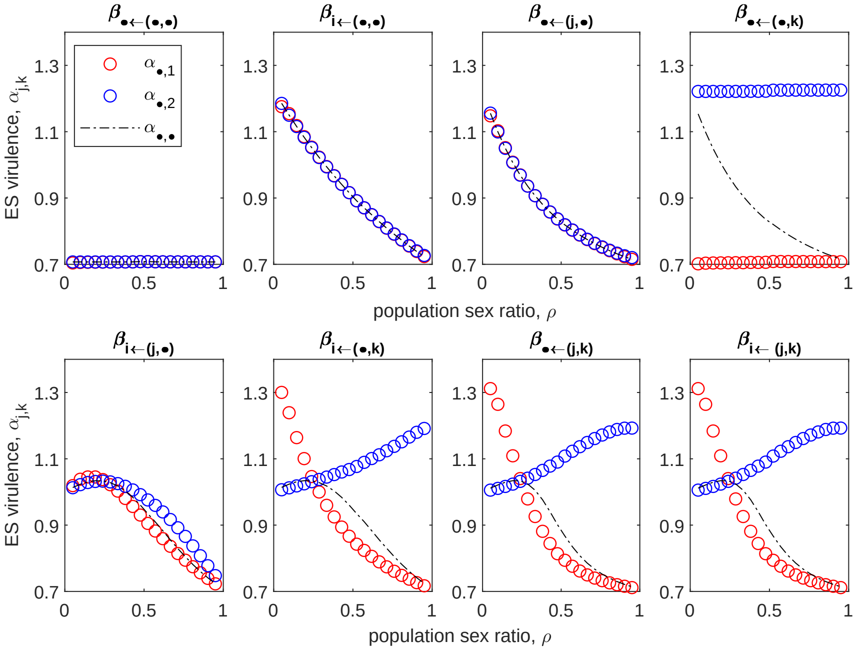

Supplementary Figure 2: Evolution of origin-specific virulence in one population. Each subplot corresponds to a different transmission dependence (indicated above the subplot). The red and blue circles are the origin-specific ES virulence for pathogens originating from either hosts of sex 1 (red) or sex 2 (blue). The dashed black line is the no plasticity ES virulence for comparison. The influx of susceptible hosts of sex 1, Λ_1, and sex 2, Λ_2, satisfies $\Lambda_1/\Lambda_2 = \rho/(1-\rho)$. Thus when $\rho < 1/2$, sex 2 is more abundant whereas when $\rho > 1/2$, sex 1 is more abundant.

then $\alpha^*_{\bullet,1} \geq \alpha^*_{\bullet,2}$. That is, if pathogens who previously infected hosts of sex 2 are more transmissible, then pathogens previously infecting hosts of sex 1 will be more virulent. This can be shown analytically: using a computer algebra package (again, we used Maple[6], the equilibrium population densities in the one population version of (1) can be shown to satisfy

$$\frac{\bar{S}_1}{\bar{S}_2} = \frac{\bar{x}_{11}}{\bar{x}_{21}} = \frac{\bar{x}_{12}}{\bar{x}_{22}} = \frac{\Lambda_1}{\Lambda_2}, \quad \frac{\bar{x}_{21}}{\bar{x}_{12}} = \frac{\alpha_{\bullet,2} + \mu + \gamma}{\alpha_{\bullet,1} + \mu + \gamma} \quad \text{and} \quad \bar{S}_2 = \frac{\Lambda_2}{\sum_i \Lambda_i \frac{\beta_{\bullet\leftarrow(\bullet,i)}}{\alpha_{\bullet,i}+\mu+\gamma}}.$$

Using these relations and (8) in the gradient of selection for virulence trait $\alpha_{\bullet,k}$, equa-

tion (6), gives

$$\lambda'_{\mathrm{O}(k)}(\alpha_{\bullet,k}) \propto \frac{\mathrm{d}\beta_{\bullet\leftarrow(\bullet,k)}}{\mathrm{d}\alpha_{\bullet,k}} - \frac{\beta_{\bullet\leftarrow(\bullet,k)}(\alpha_{\bullet,k})}{\alpha_{\bullet,k}+\mu+\gamma}.$$

Hence using transmission function (2), the ES virulence is $\alpha^*_{\bullet,k} = \sqrt{\theta_{\bullet\leftarrow(\bullet,k)}(\mu+\gamma)}$ and so as $\theta_{\bullet\leftarrow(\bullet,k)}$ increases, $\beta_{\bullet\leftarrow(\bullet,k)}(\alpha)$ decreases, and the ES virulence $\alpha^*_{\bullet,k}$ increases. In fact, more generally we would expect that if

$$\beta_{i\leftarrow(j,2)}(\alpha) \geq \beta_{i\leftarrow(j,1)}(\alpha) \quad \text{for all } i, j$$

then $\alpha^*_{\bullet,1} \geq \alpha^*_{\bullet,2}$. The logic here is as before and is a standard result of virulence evolution under a transmission-virulence trade-off: as the relative rate of increase in transmission increases, the ES virulence decreases see e.g.,[4,5].

(2) Transmission does not depend upon host-of-origin. From our earlier analysis, we know that origin-specific virulence can only invade if transmission depends upon both the sex of current host and the sex of susceptible host, $\beta_{i\leftarrow(j,\bullet)}(\alpha)$. From (14) and (15) the necessary and sufficient conditions for $\alpha^*_{\bullet,1} > \alpha^*_{\bullet,2}$ is that

$$\mathrm{sign}\left(\beta_{2\leftarrow(1,\bullet)}\beta_{1\leftarrow(2,\bullet)} - \beta_{1\leftarrow(1,\bullet)}\beta_{2\leftarrow(2,\bullet)}\right) = \mathrm{sign}\left(\left.\frac{\mathrm{d}\mathcal{R}_{2,\bullet}}{\mathrm{d}\alpha}\right|_{\alpha=\alpha^*_{\bullet,\bullet}}\right). \tag{16}$$

Using a computer algebra package[6], it can be shown that

$$\frac{\mathrm{d}\mathcal{R}_{2,\bullet}}{\mathrm{d}\alpha} \propto \frac{\beta_{2\leftarrow(1,\bullet)}}{\bar{x}_{2,1}+\bar{x}_{2,2}}\frac{\mathrm{d}}{\mathrm{d}\alpha}\left[\frac{\beta_{1\leftarrow(2,\bullet)}}{\mu+\gamma+\alpha}\right]_{\alpha=\alpha^*_{\bullet,\bullet}} + \frac{\beta_{1\leftarrow(2,\bullet)}}{\bar{x}_{1,1}+\bar{x}_{1,2}}\frac{\mathrm{d}}{\mathrm{d}\alpha}\left[\frac{\beta_{2\leftarrow(2,\bullet)}}{\mu+\gamma+\alpha}\right]_{\alpha=\alpha^*_{\bullet,\bullet}} \tag{17}$$

where proportionality is up to multiplication by a positive constant. From inspection of (15) and (17), we see that the two factors determining whether or not $\alpha^*_{\bullet,1} > \alpha^*_{\bullet,2}$ are the relationship between the cross-sex and same-sex transmission functions, given in (15), and the population composition in (17) which is primarily determined by the specification of Λ_1 and Λ_2, which controls the influx of susceptibles of different sexes

to the population.

Let us consider a concrete example to show the influence of both population composition and transmission. Suppose $\beta_{2\leftarrow(1,\bullet)} = \beta_{1\leftarrow(2,\bullet)}$ and $\beta_{1\leftarrow(1,\bullet)} = \beta_{2\leftarrow(2,\bullet)}$ that is, cross-sex and same-sex transmission are independent of direction of transmission. Then, at the ES virulence for no plasticity, $\alpha^*_{\bullet,\bullet}$, we would expect that

$$\frac{\mathrm{d}}{\mathrm{d}\alpha}\left[\frac{\beta_{1\leftarrow(2,\bullet)}}{\mu+\gamma+\alpha}\right]_{\alpha=\alpha^*_{\bullet,\bullet}} \quad \text{and} \quad \frac{\mathrm{d}}{\mathrm{d}\alpha}\left[\frac{\beta_{2\leftarrow(2,\bullet)}}{\mu+\gamma+\alpha}\right]_{\alpha=\alpha^*_{\bullet,\bullet}} \tag{18}$$

have opposite sign. The reason for this is that if they shared the same sign we could not be at the ES virulence, as selection would either favour decreased (if both negative) or increased (if both positive) virulence. For the transmission function (2) the virulence at which the quantities in (18) change sign is an increasing function of $\theta_{i\leftarrow(j,\bullet)}$.

Therefore if cross-sex transmission occurs more readily than same-sex transmission, we must have

$$\theta_{2\leftarrow(1,\bullet)} = \theta_{1\leftarrow(2,\bullet)} < \theta_{1\leftarrow(1,\bullet)} = \theta_{2\leftarrow(2,\bullet)}, \tag{19}$$

and so we would expect

$$\frac{\mathrm{d}}{\mathrm{d}\alpha}\left[\frac{\beta_{1\leftarrow(2,\bullet)}}{\mu+\gamma+\alpha}\right]_{\alpha=\alpha^*_{\bullet,\bullet}} < 0 \quad \text{and} \quad \frac{\mathrm{d}}{\mathrm{d}\alpha}\left[\frac{\beta_{2\leftarrow(2,\bullet)}}{\mu+\gamma+\alpha}\right]_{\alpha=\alpha^*_{\bullet,\bullet}} > 0. \tag{20}$$

If we use the information in (20) in (17), we see that the sign of (17) is determined by the population sex ratio, that is, $\bar{x}_{1,1} + \bar{x}_{1,2}$ and $\bar{x}_{2,1} + \bar{x}_{2,2}$, which in turn is controlled by the influx of susceptibles of sex 1 and 2, Λ_1 and Λ_2. Let $\Lambda_1 = \rho\Lambda$ and $\Lambda_2 =$

$(1-\rho)\Lambda$. Then

$$\begin{aligned}
&\text{if } \rho < 1/2 \text{ then} \quad \bar{x}_{1,1} + \bar{x}_{1,2} < \bar{x}_{2,1} + \bar{x}_{2,2} \quad \text{and } \alpha^*_{\bullet,1} > \alpha^*_{\bullet,2} \\
&\text{if } \rho = 1/2 \text{ then} \quad \bar{x}_{1,1} + \bar{x}_{1,2} = \bar{x}_{2,1} + \bar{x}_{2,2} \quad \text{and } \alpha^*_{\bullet,1} = \alpha^*_{\bullet,2} \\
&\text{if } \rho > 1/2 \text{ then} \quad \bar{x}_{1,1} + \bar{x}_{1,2} > \bar{x}_{2,1} + \bar{x}_{2,2} \quad \text{and } \alpha^*_{\bullet,1} < \alpha^*_{\bullet,2}
\end{aligned} \tag{21}$$

The logic for the relations in (21) is straightforward: if $\rho < 1/2$, then $\bar{x}_{1,1} + \bar{x}_{1,2} < \bar{x}_{2,1} + \bar{x}_{2,2}$, and so the second term in (17) is a more important contributor to the sign of (17); since by (20) it is positive, and since from (19) we will have (15) positive, we can conclude that $\alpha^*_{\bullet,1} > \alpha^*_{\bullet,2}$. Similar logic follows when $\rho > 1/2$. When $\rho = 1/2$, there is no difference between subpopulations and so origin-specific virulence will not invade. If instead same-sex transmission occurs more readily than cross-sex transmission (i.e., inequality (19) is reversed), the same argument follows and relations (21) still hold. In Figure 3 we provide numerical support for these predictions.

If we relax the assumption that $\theta_{2\leftarrow(1,\bullet)} = \theta_{1\leftarrow(2,\bullet)}$ and $\theta_{1\leftarrow(1,\bullet)} = \theta_{2\leftarrow(2,\bullet)}$, then we need to rely upon numerical results. Numerical simulations with $\rho = 1/2$ broadly confirm the predictions of our analysis, and underline the importance of two factors arising from our analysis. The first factor is a comparison of the geometric mean of cross-sex and same-sex transmission, that is, whether $\sqrt{\beta_{2\leftarrow(1,\bullet)}\beta_{1\leftarrow(2,\bullet)}}$ is greater or less than $\sqrt{\beta_{2\leftarrow(2,\bullet)}\beta_{1\leftarrow(1,\bullet)}}$, which arises from the left-hand side of (16). The second factor is a comparison of the arithmetic mean of transmission conditioned on current host, that is, whether $\beta_{1\leftarrow(2,\bullet)} + \beta_{2\leftarrow(2,\bullet)}$ is greater or less than $\beta_{1\leftarrow(1,\bullet)} + \beta_{2\leftarrow(1,\bullet)}$, which arises from consideration of (17).

Note 4.2 Two subpopulations

In the two subpopulation model we are primarily interested in situations in which origin-specific virulence cannot invade in the one population model, in particular, when

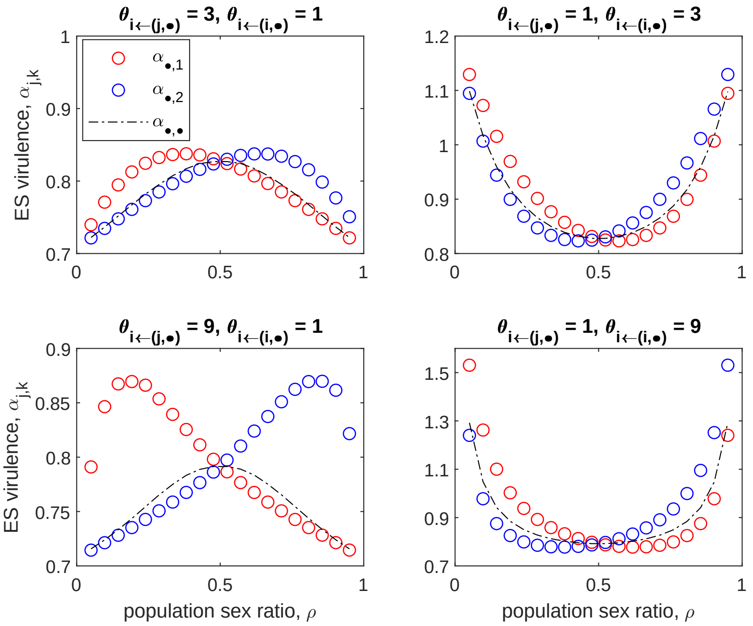

Supplementary Figure 3: Evolution of origin-specific virulence in one population when transmission depends upon both current and future host, $\beta_{i\leftarrow(j,\bullet)}$ and $\beta_{1\leftarrow(1,\bullet)}(\alpha) = \beta_{2\leftarrow(2,\bullet)}(\alpha)$ and $\beta_{1\leftarrow(2,\bullet)}(\alpha) = \beta_{2\leftarrow(1,\bullet)}(\alpha)$. The influx of susceptible hosts of sex 1, Λ_1, and sex 2, Λ_2, satisfies $\Lambda_1/\Lambda_2 = \rho/(1-\rho)$. Thus when $\rho < 1/2$, sex 2 is more abundant whereas when $\rho > 1/2$, sex 1 is more abundant. In fact ρ is the critical predictor of the relationship between $\alpha_{\bullet,1}$ and $\alpha_{\bullet,2}$: when hosts of sex 2 are more abundant, $\alpha_{\bullet,1} > \alpha_{\bullet,2}$, whereas when hosts of sex 1 are more abundant, $\alpha_{\bullet,1} < \alpha_{\bullet,2}$. This holds irrespective of whether pathogens are more or less transmissible cross-sex versus same-sex. Note that $\theta_{i\leftarrow(j,\bullet)} = \theta_{1\leftarrow(2,\bullet)} = \theta_{2\leftarrow(1,\bullet)}$ and $\theta_{i\leftarrow(i,\bullet)} = \theta_{1\leftarrow(1,\bullet)} = \theta_{2\leftarrow(2,\bullet)}$.

transmission is either of the form $\beta_{i\leftarrow(\bullet,\bullet)}$ or $\beta_{\bullet\leftarrow(j,\bullet)}$. Here we have to rely upon numerical results (Fig. 4). We consider the evolution of origin-specific virulence when the subpopulations differ based upon either sex-ratio or contact network. For the sake of comparison with the one population results, we also consider the case in which transmission depends upon both the sex of the susceptible host and sex of current host in two subpopulations with differences in sex-ratio. The intuitive explanation for these results is provided in the main text.

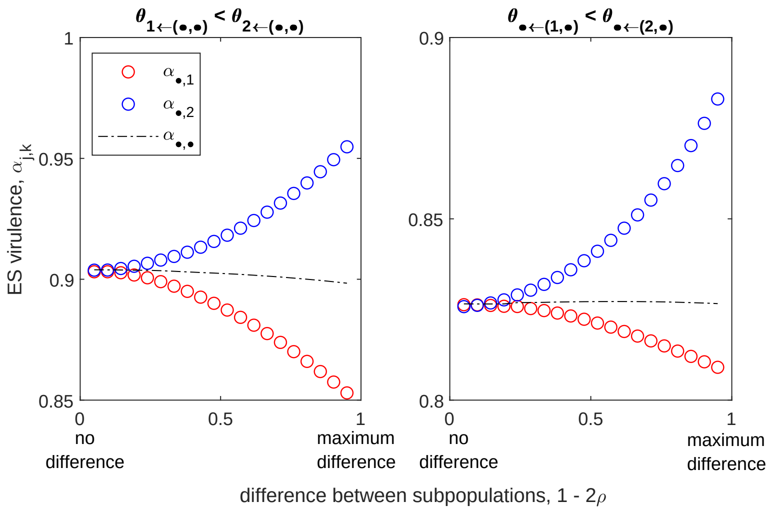

Supplementary Figure 4: Evolution of origin-specific virulence in two subpopulations. Our focus is when origin-specific virulence is predicted to not invade in the one population model: $\beta_{i\leftarrow(\bullet,\bullet)}$ and $\beta_{\bullet\leftarrow(j,\bullet)}$. The difference between subpopulations is controlled by the influx of susceptibles; in particular, $\Lambda_1^A = \Lambda_2^B$, $\Lambda_2^A = \Lambda_1^B$ with $\Lambda_1^A/\Lambda_1^B = \rho/(1-\rho)$. We restrict $\rho \in [0, 1/2]$, so the difference between subpopulations is $\Lambda_1^B - \Lambda_1^A = 1 - 2\rho$. When $\rho = 1/2$, $1 - 2\rho = 0$ and the subpopulations are identical. When $\rho = 0$, $1 - 2\rho = 1$ and the subpopulations are maximally different. All subplots used transmission function (2) and the relationship between the choice of $\theta_{i\leftarrow(j,k)}$ parameters is shown above each subplot.

Note 5 Origin-and-sex-specific virulence

We define origin-and-sex-specific virulence as when there is a pattern of virulence that cannot be replicated by either sex- or origin-specific virulence alone.

Note 5.1 One population

Note 5 When does origin-and-sex-specific virulence invade?

In the one population model, only when transmission depends explicitly upon both host-of-origin and current host will origin-and-sex-specific virulence invade. This follows directly from the conditions under which sex-specific and origin-specific virulence can individually invade.

Note 5 When does cross-sex transmission lead to greater virulence than same-sex transmission?

If transmission does not depend upon sex of the susceptible host, $\beta_{\bullet\leftarrow(j,k)}(\alpha)$, then we would expect that the ordering of the origin-and-sex-specific ES virulence will be dictated by the ordering relationship amongst the transmission functions. In particular, (j,k) combinations with greater transmission favour a lower ES virulence $\alpha^*_{j,k}$. Thus cross-sex transmission will lead to higher virulence than same-sex transmission if

$$\beta_{\bullet\leftarrow(2,2)}(\alpha),\ \beta_{\bullet\leftarrow(1,1)}(\alpha) > \beta_{\bullet\leftarrow(2,1)}(\alpha),\ \beta_{\bullet\leftarrow(1,2)}(\alpha).$$

For example, if $\beta_{\bullet\leftarrow(2,2)} > \beta_{\bullet\leftarrow(1,1)} > \beta_{\bullet\leftarrow(2,1)} > \beta_{\bullet\leftarrow(1,2)}$, the origin-and-sex-specific ES virulence will satisfy $\alpha^*_{1,2} > \alpha^*_{2,1} > \alpha^*_{1,1} > \alpha^*_{2,2}$. Moreover, when transmission does not depend upon susceptible host, the ES virulence $\alpha_{j,k}$ is the solution of

$$\frac{d\beta_{\bullet\leftarrow(j,k)}}{d\alpha_{j,k}} - \frac{\beta_{\bullet\leftarrow(j,k)}(\alpha_{j,k})}{\alpha_{j,k}+\mu+\gamma} = 0, \tag{22}$$

so using the transmission function (2), $\alpha^*_{j,k} = \sqrt{\theta_{\bullet\leftarrow(j,k)}(\mu+\gamma)}$. This can be verified numerically (not shown) by checking that

$$\frac{v_{jk}}{v_{1j}\bar{S}_1 + v_{2j}\bar{S}_2} = \frac{\beta_{\bullet\leftarrow(j,k)}(\alpha_{j,k})}{\alpha_{j,k}+\mu+\gamma};$$

when this holds it is clear $\lambda'_{j,k}(\alpha_{j,k})$ is proportional to the left-hand side of (22) and so the evolution of the $\alpha_{j,k}$ is a maximization problem. Thus when transmission does not depend upon susceptible host sex, origin-and-sex-specific virulence evolves to maximize pathogen fitness for each infection history independently.

Note 5.2 Two subpopulations

The extension to two subpopulations is similar. In this case, we are interested in the situation where transmission has no dependence upon host-of-origin, $\beta_{i\leftarrow(j,\bullet)}(\alpha)$, as

these are situations in which origin-and-sex-specific virulence does not invade in the one population model. Our objective is to investigate when cross-sex transmission leads to greater virulence than same-sex transmission and when it will not.

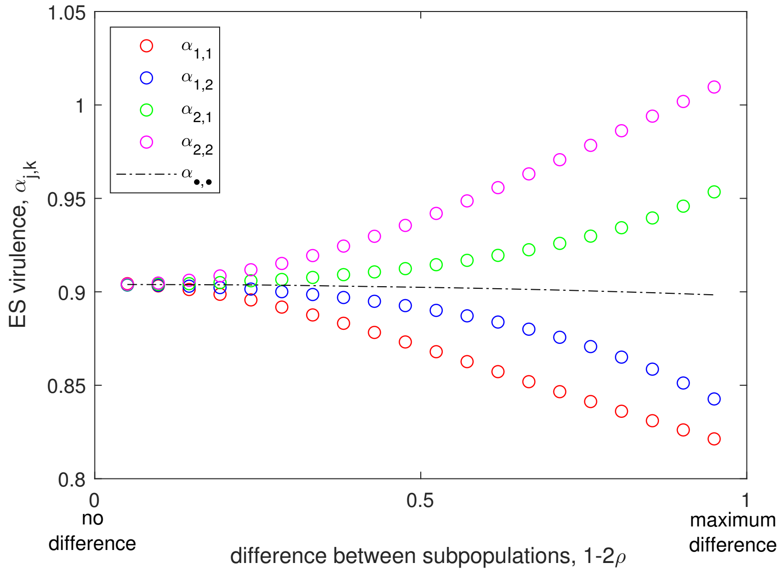

Supplementary Figure 5: Evolution of origin-and-sex-specific virulence in two subpopulations differing based upon sex ratio with $\beta_{i\leftarrow(\bullet,\bullet)}$. Here we have assumed it is easier to transmit to sex 1 hosts then sex 2 hosts, $\theta_{2\leftarrow(\bullet,\bullet)} > \theta_{1\leftarrow(\bullet,\bullet)}$, and so pathogens are more virulent in hosts of sex 2. Here $\Lambda_1^A = \Lambda_2^B$, $\Lambda_2^A = \Lambda_1^B$ with $\Lambda_1^A/\Lambda_1^B = \rho/(1-\rho)$. We restrict $\rho \in [0, 1/2]$, so the difference between subpopulations is $\Lambda_1^B - \Lambda_1^A = 1 - 2\rho$. When $\rho = 1/2$, $1 - 2\rho = 0$ and the subpopulations are identical. When $\rho = 0$, $1 - 2\rho = 1$ and the subpopulations are maximally different.

First, suppose the only difference between the subpopulations is sex ratio, that is, subpopulation A consists primarily of sex 2 hosts and subpopulation B consists primarily of sex 1 hosts. Here we set $\Lambda_1^\ell = \Lambda\rho_\ell$, $\Lambda_2^\ell = \Lambda(1-\rho_\ell)$, with $0 \leq \rho_A < 1/2$ and $1/2 < \rho_B \leq 1$, and let $c^\ell_{i\leftrightarrow j} = 1$. There are two cases of interest: transmission depends only upon susceptible host, $\beta_{i\leftarrow(\bullet,\bullet)}$ and transmission depends upon both current and susceptible host, $\beta_{i\leftarrow(j,\bullet)}$.

i. Transmission only depends upon susceptible host sex, $\beta_{i\leftarrow(\bullet,\bullet)}(\alpha)$. Without loss of generality, let $\beta_{1\leftarrow(\bullet,\bullet)}(\alpha) > \beta_{2\leftarrow(\bullet,\bullet)}(\alpha)$, that is, all else being equal it is easier to trans-

mit to sex 1 than sex 2. For example, sex 1 hosts may engage in riskier behaviour and this occurs irrespective of subpopulation. Then the ES virulence ordering is $\alpha^*_{2,2} > \alpha^*_{2,1} > \alpha^*_{1,2} > \alpha^*_{1,1}$ (Fig. 5). Thus only one sex experiences greater virulence when transmission is cross- rather than same-sex. The intuition behind this is the $\alpha^*_{j,k}$ reflects the pathogen's 'confidence' that it is in currently in a particular subpopulation, based upon infection history.

ii. Transmission depends upon both current and susceptible host sex, $\beta_{i\leftarrow(j,\bullet)}(\alpha)$. To make analytic progress, consider the situation in which $\Lambda^A_1 = \Lambda^B_2 = \Lambda\rho$, $\Lambda^A_2 = \Lambda^B_1 = \Lambda(1-\rho)$, $\alpha_{\bullet,\bullet}$, $\beta_{1\leftarrow(2,\bullet)}(\alpha) = \beta_{2\leftarrow(1,\bullet)}(\alpha)$, $\beta_{1\leftarrow(1,\bullet)}(\alpha) = \beta_{2\leftarrow(2,\bullet)}(\alpha)$. Under these conditions, at equilibrium

$$\bar{S}^A_i = \bar{S}^B_j, \quad \bar{x}^A_{i,j} = \bar{x}^B_{j,i} \quad \text{and} \quad x^A_{i,i} = x^B_{j,j} \quad \text{for } i \neq j. \tag{23}$$

Moreover, the left eigenvector takes the form

$$\mathbf{v} = [v^B_{2,\bullet}, v^B_{2,\bullet}, v^B_{1,\bullet}, v^B_{1,\bullet}, v^B_{1,\bullet}, v^B_{1,\bullet}, v^B_{2,\bullet}, v^B_{2,\bullet}], \text{ with } \frac{v^B_{2,\bullet}}{v^B_{1,\bullet}} = \frac{\sigma + \beta_{1\leftarrow(2,\bullet)}\bar{S}^B_1}{\mu + \gamma + \alpha + \sigma}. \tag{24}$$

It follows that $\frac{\mathrm{d}\mathcal{R}^\ell_{j,k}}{\mathrm{d}\alpha} = \frac{\mathrm{d}\mathcal{R}^\ell_{j,\bullet}}{\mathrm{d}\alpha}$. Now suppose the virulence in the population is $\alpha_{1,1} = \alpha_{1,2} = \alpha^*_{1,1}$ and $\alpha_{2,1} = \alpha_{2,2} = \alpha^*_{2,2}$, where $\alpha^*_{1,1}$ satisfies $\lambda'_{1,1}(\alpha^*_{1,1}) = 0$ and $\alpha^*_{2,2}$ satisfies $\lambda'_{2,2}(\alpha^*_{2,2}) = 0$. Because of our choice of demographic parameters, it is apparent that $\alpha^*_{1,1} = \alpha^*_{2,2}$, and so we will use α^* to indicate this value. Using the relation

$\lambda'_{1,1}(\alpha^*) = \lambda'_{2,2}(\alpha^*) = 0$, and the results (23) and (24), we can write

$$\lambda'_{j,k}(\alpha^*) \propto \sum_i \left(\bar{x}^B_{k,i} - \bar{x}^B_{j,i}\right) \left(\sum_i \bar{x}^B_{j,i} \frac{\mathrm{d}}{\mathrm{d}\alpha}\left[\frac{\beta_{1\leftarrow(1,\bullet)}}{\mu+\gamma+\alpha+\sigma}\right]_{\alpha=\alpha^*} + \sum_i \bar{x}^B_{k,i} \frac{\mathrm{d}}{\mathrm{d}\alpha}\left[\frac{\beta_{1\leftarrow(2,\bullet)}}{\mu+\gamma+\alpha+\sigma}\right]_{\alpha=\alpha^*} - \frac{\sigma\sum_i \bar{x}^B_{k,i}}{(\mu+\gamma+\alpha+\sigma)^2}\left[\frac{\mathrm{d}\beta_{1\leftarrow(1,\bullet)}}{\mathrm{d}\alpha}\right]_{\alpha=\alpha^*} + \frac{\sigma\sum_i \bar{x}^B_{j,i}}{(\mu+\gamma+\alpha+\sigma)^2}\left[\frac{\mathrm{d}\beta_{1\leftarrow(2,\bullet)}}{\mathrm{d}\alpha}\right]_{\alpha=\alpha^*}\right), \; j \neq k. \tag{25}$$

We are interested in the conditions under which both $\lambda'_{1,2}(\alpha^*) > 0$ and $\lambda'_{2,1}(\alpha^*) > 0$. Suppose σ becomes very small. Then (25) can be approximated by

$$\lambda'_{j,k}(\alpha^*) \approx \sum_i \left(\bar{x}^B_{k,i} - \bar{x}^B_{j,i}\right) \left(\sum_i \bar{x}^B_{j,i} \frac{\mathrm{d}}{\mathrm{d}\alpha}\left[\frac{\beta_{1\leftarrow(1,\bullet)}}{\mu+\gamma+\alpha}\right]_{\alpha=\alpha^*} + \sum_i \bar{x}^B_{k,i} \frac{\mathrm{d}}{\mathrm{d}\alpha}\left[\frac{\beta_{1\leftarrow(2,\bullet)}}{\mu+\gamma+\alpha}\right]_{\alpha=\alpha^*}\right). \tag{26}$$

Following similar logic as before, because $\alpha^*_{1,1} = \alpha^*_{2,2} = \alpha^*$, at this ES virulence we must have the conditions in (18) to hold (recall $\beta_{1\leftarrow(1,\bullet)} = \beta_{2\leftarrow(2,\bullet)}$). Now focus upon the transmission function given by (2). It was shown earlier that if (say)

$$\frac{\mathrm{d}}{\mathrm{d}\alpha}\left[\frac{\beta_{1\leftarrow(2,\bullet)}}{\mu+\gamma+\alpha}\right]_{\alpha=\alpha^*} < 0$$

then $\beta_{1\leftarrow(2,\bullet)}(\alpha^*) > \beta_{1\leftarrow(1,\bullet)}(\alpha^*)$ (and vice-versa). From (26), if $\beta_{1\leftarrow(k,\bullet)}(\alpha^*) > \beta_{1\leftarrow(j,\bullet)}(\alpha^*)$, cross-sex transmission will lead to greater virulence than same-sex transmission if

$$\frac{\sum_i \bar{x}^B_{j,i}}{\sum_i \bar{x}^B_{k,i}} > -\frac{\frac{\mathrm{d}}{\mathrm{d}\alpha}\left[\frac{\beta_{1\leftarrow(k,\bullet)}}{\mu+\gamma+\alpha}\right]_{\alpha=\alpha^*}}{\frac{\mathrm{d}}{\mathrm{d}\alpha}\left[\frac{\beta_{1\leftarrow(j,\bullet)}}{\mu+\gamma+\alpha}\right]_{\alpha=\alpha^*}} > \frac{\sum_i \bar{x}^B_{k,i}}{\sum_i \bar{x}^B_{j,i}}$$

This can only be satisfied if $j = 2$, $k = 1$, that is, $\beta_{1\leftarrow(1,\bullet)}(\alpha) > \beta_{1\leftarrow(2,\bullet)}(\alpha)$ (there is a contradiction when $j = 1$, $k = 2$). Hence if the subpopulations are sufficiently different, cross-sex transmission will lead to higher (resp. lower) virulence than

same-sex transmission if same-sex transmission occurs more readily than cross-sex transmission (Fig. 6).

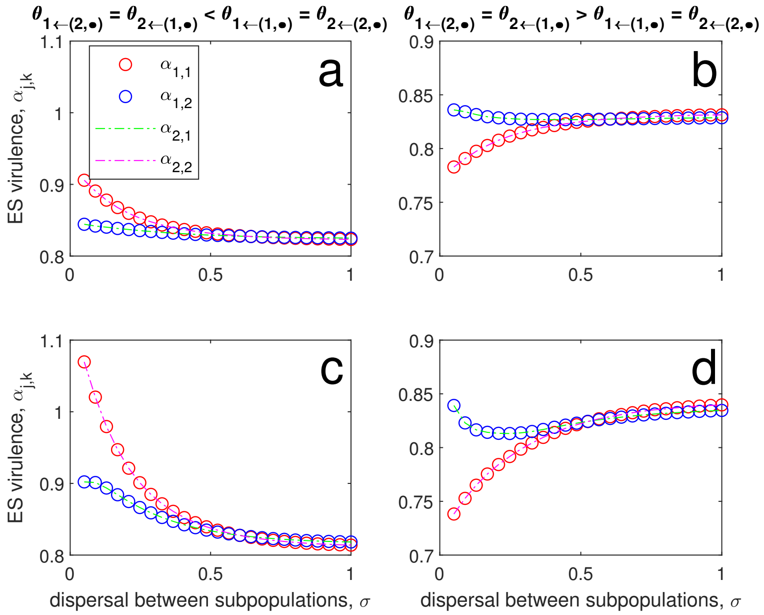

Supplementary Figure 6: Evolution of origin-and-sex-specific virulence in two subpopulations differing based upon sex ratio, $\Lambda_1^A = \Lambda_2^B$ and $\Lambda_2^A = \Lambda_1^B$ with $\Lambda_1^A/\Lambda_1^B = \rho/(1-\rho)$. In subplots **a** and **c** (resp. **b** and **d**), the pathogen is more (resp. less) transmissible cross-sex rather than same-sex. In subplots **a** and **b**, the subpopulations are more similar ($\rho = 0.25$) than in subplots **c** and **d** ($\rho = 0.05$). Note by symmetry, the ES virulence satisfies $\alpha_{1,2} = \alpha_{2,1}$ and $\alpha_{1,1} = \alpha_{2,2}$.

Indeed, numerical results show the the conclusions derived when $\beta_{i\leftarrow(j,\bullet)}(\alpha)$ hold more generally. In particular, if we allow for

$$\beta_{1\leftarrow(1,\bullet)}(\alpha) \neq \beta_{2\leftarrow(2,\bullet)}(\alpha) \quad \text{and} \quad \beta_{1\leftarrow(2,\bullet)}(\alpha) \neq \beta_{2\leftarrow(1,\bullet)}(\alpha),$$

we will still arrive at the prediction that if same-sex transmission occurs more readily than cross-sex transmission, cross-sex transmitted pathogens are more virulent than same-sex transmitted pathogens. This occurs irrespective of whether the difference between subpopulations is sex-ratio or contact network.

References

1. Otto, S. P. & Day, T. *A Biologist's Guide to Mathematical Modeling in Ecology and Evolution* (Princeton University Press, 2007).

2. Taylor, P. D. & Frank, S. A. How to make a kin selection model. *J. Theor. Biol.* **180**, 27–37 (1996).

3. MATLAB. *version 9.6.0 (R2019a)* (The MathWorks Inc., Natick, Massachusetts, 2019).

4. Anderson, R. & May, R. Coevolution of hosts and parasites. *Parasitology* **85**, 411–426 (1982).

5. Frank, S. A. Models of parasite virulence. *Q. Rev. Biol* **71**, 37–78 (1996).

6. Maple. *2019 Release* (Maplesoft, Waterloo, Ontario, Canada, 2019).
